# Cytokine Profiles of Severe Influenza Virus-Related Complications in Children

**DOI:** 10.3389/fimmu.2017.01423

**Published:** 2017-11-06

**Authors:** Andrew Fiore-Gartland, Angela Panoskaltsis-Mortari, Anna A. Agan, Anushay J. Mistry, Paul G. Thomas, Michael A. Matthay, PALISI PICFlu Investigators, Ronald C. Sanders, Tomer Hertz, Adrienne G. Randolph

**Affiliations:** ^1^Vaccine and Infectious Disease Division, Fred Hutchinson Cancer Research Center, Seattle, WA, United States; ^2^Department of Pediatrics, Bone Marrow Transplantation, Pulmonary and Critical Care Medicine, University of Minnesota, Minneapolis, MN, United States; ^3^Department of Anesthesiology, Perioperative, and Pain Medicine, Boston Children’s Hospital, Boston, MA, United States; ^4^Department of Immunology, St. Jude Children’s Research Hospital, Memphis, TN, United States; ^5^Department of Medicine, University of California, San Francisco, San Francisco, CA, United States; ^6^Department of Anesthesia, University of California, San Francisco, San Francisco, CA, United States; ^7^The Shraga Segal Department of Microbiology, Immunology and Genetics, Ben-Gurion University of the Negev, Be’er-Sheva, Israel; ^8^National Institute for Biotechnology in the Negev, Ben-Gurion University of the Negev, Be’er-Sheva, Israel; ^9^Department of Anaesthesia, Harvard Medical School, Boston, MA, United States; ^10^Department of Pediatrics, Harvard Medical School, Boston, MA, United States

**Keywords:** influenza, cytokine, acute lung injury, septic shock, inflammation

## Abstract

**Rationale:**

Effective immunomodulatory therapies for children with life-threatening “cytokine storm” triggered by acute influenza infection are lacking. Understanding the immune profiles of children progressing to severe lung injury and/or septic shock could provide insight into pathogenesis.

**Objectives:**

To compare the endotracheal and serum cytokine profiles of children with influenza-related critical illness and to identify their associations with severe influenza-associated complications.

**Methods:**

Children with influenza-related critical illness were enrolled across 32 hospitals in development (*N* = 171) and validation (*N* = 73) cohorts (December 2008 through May 2016). Concentrations of 42 cytokines were measured in serum and endotracheal samples and clustered into modules of covarying cytokines. Relative concentrations of cytokines and cytokine modules were tested for associations with acute lung injury (ALI), shock requiring vasopressors, and death/ECMO.

**Measurements and main results:**

Modules of covarying cytokines were more significantly associated with disease severity than individual cytokines. In the development cohort, increased levels of a serum module containing IL6, IL8, IL10, IP10, GCSF, MCP1, and MIP1α [shock odds ratio (OR) = 3.37, family-wise error rate (FWER) *p* < 10^−4^], and decreased levels of a module containing EGF, FGF2, SCD40L, and PAI-1 (shock OR = 0.43, FWER *p* = 0.002), were both associated with ALI, shock, and death-ECMO independent of age and bacterial coinfection. Both of these associations were confirmed in the validation cohort. Endotracheal and serum cytokine associations differed markedly and were differentially associated with clinical outcomes.

**Conclusion:**

We identified strong positive and negative associations of cytokine modules with the most severe influenza-related complications in children, providing new insights into the pathogenesis of influenza-related critical illness in children. Effective therapies may need to target mediators of both inflammation and repair.

## Introduction

Influenza virus is one of the leading causes of hospitalizations and deaths in children (1). Influenza-related lower respiratory tract infection that progresses to acute lung injury (ALI) with profound hypoxia and respiratory failure, or to systemic inflammation leading to circulatory collapse requiring vasopressors (i.e., septic shock), is often deadly. Patients developing both ALI and shock have the highest mortality. Many deaths are related to superinfection with bacteria that colonize the upper airway, such as *Staphylococcus aureus* (2). Understanding the influenza-associated immune phenotype of patients with ALI and shock could facilitate the development of prognostic biomarkers or targeted therapies to terminate harmful inflammation and enhance mediators of recovery (3).

Studies of infection in mice and humans with particularly virulent influenza A viruses, including pandemic 2009 H1N1 (pH1N1) and avian H7N9 and H5N1, have yielded important insights into the proinflammatory factors involved in host response and pathogenesis (4–16). Unfortunately, data in children with severe influenza are limited. In a small cohort of critically ill children and young adults, we previously reported associations between high levels of individual cytokines circulating in the bloodstream (IL6, IL8, IP10, GMCSF, MCP1, and MIP1α) with fatal influenza infection (17). In pediatric outpatients infected with influenza, we also identified a proinflammatory cytokine profile that was associated with symptom severity after controlling for viral load and age (18). Because these children had high intracompartment (e.g., blood, nasopharynx) correlation among cytokines (18), we hypothesized that overall levels of immune activation drive absolute cytokine concentrations. A high level of generalized immune activation could obscure identification of cytokines that are relatively deficient. Technical artifacts, such as sampling variability introduced by methods for respiratory sample collection, can also affect absolute cytokine concentration. We employed linear regression in a novel context to adjust for the overall concentration of cytokines and estimate each cytokines relative concentration in the sample. Focusing on relative, as opposed to absolute, cytokine concentration helps control for these artifacts and reveals a more complex signaling pattern.

Previous work by our group and others tested for associations of individual cytokines with illness severity (17–19), however, a viral infection induces a complex cytokine response, both at the site of infection and in the periphery. We hypothesized that patterns of cytokine covariation would provide novel insights into the pathophysiology of disease severity-related phenotypes. Therefore, in this study, we employed a modular approach to cytokine analysis that leverages covariation and intrinsic redundancy to identify patterns of immune signaling that can be evaluated for their associations with disease. With this approach, our primary aim was to characterize the cytokine profiles of children with life-threatening and fatal influenza-related complications including ALI, septic shock, and death or receipt of extracorporeal life support (ECMO).

## Materials and Methods

We enrolled children with suspected influenza critical illness at 35 hospitals (Table S13 in Supplementary Material). Children with underlying medical conditions predisposing to severe influenza infection (17) were excluded (see Table S1 in Supplementary Material for detailed inclusion/exclusion criteria). The development cohort included patients enrolled December 2008 to April 2014 with a positive influenza test (DFA, PCR, or culture). The validation cohort included influenza virus positive children enrolled November 2015 to May 2015 and patients with positive rapid influenza tests December 2008 to April 2014 without PCR confirmation. We excluded children included in prior reports (17). The Institutional Review Boards at each site approved the study and written informed consent was obtained from at least one parent or guardian.

Samples of blood and endotracheal aspirates (if intubated) were provided; BSL2 and universal precautions were taken with all samples. Samples were provided at enrollment (mostly within 24 h of intensive care unit admission). The pediatric risk of mortality (PRISM) III score was used for admission severity. ALI was defined by American-European Consensus Conference criteria (20). We quantified vasoactive agent use with the cardiovascular sepsis-related organ failure assessment (CV-SOFA) score as described in the Presentation S1 in Supplementary Material (21). Septic shock was defined as CV-SOFA ≥ 2 in the cohort. The concentration of 42 cytokines were measured in duplicate by the University of Minnesota Cytokine Reference Laboratory using standard ELISA as well as bead-based Luminex multiplex assays (see Presentation S1 in Supplementary Material). Cytokines were chosen for inclusion based upon prior reports of their association with influenza infection (3, 17, 22–24).

### Statistical and Modeling Approaches

Cytokine concentrations (pg/mL) were log-transformed for all analyses. Pearson’s correlation was used to assess cytokine covariation. To adjust for high correlation among cytokines, relative concentrations were computed from the absolute concentrations by adjusting for the mean cytokine level within each sample; the effect was that all downstream analyses of relative concentrations were independent of the mean cytokine concentrations of each sample (see Presentation S1 in Supplementary Material for details). Briefly, each cytokine variable was regressed on a vector of mean concentrations in each sample, and the residuals were taken as cytokine concentration adjusted for the mean. Cytokine modules were constructed with ET and blood stream (BS) samples separately, using hierarchical clustering on a correlation-based similarity metric; patient-level bootstrapping was employed to improve cluster reliability (25). Modules were also constructed using absolute cytokine concentrations. The score for each module and each sample was the mean concentration of cytokines in the module. This approach of using relative analyte concentrations in a module-based analysis is described in detail the Presentation S1 in Supplementary Material.

The prespecified primary analysis included evaluation of each BS and ET modules for associations with clinical complications using logistic regression, with and without adjustment for age and bacterial coinfection. An exploratory analysis followed to evaluate the associations of individual cytokines with the clinical complications. Associations of individual cytokines and modules are reported using an odds ratio (OR) and a fold-difference in concentration or module score between patients with and without the respective complication. For the primary analysis significance was based on a conservative family-wise error rate (FWER) adjusted *p* < 0.05 accounting for 182 comparisons (including modules based on relative and absolute cytokine concentrations). False-discovery rate (FDR) adjusted *q*-values are also reported. Significant modules of relative cytokines in the development cohort were tested for associations in the validation cohort, using unadjusted *p* < 0.05 as criteria for successful validation. Except where indicated, figures and text describe analyses of relative as opposed to absolute cytokine concentrations. Python code for all statistical analyses is publicly available on github: https://github.com/agartland/cycluster.

## Results

Characteristics of the 171 children with influenza critical illness in the development cohort were overall similar to those of the 76 children in the validation cohort (Table 1). Almost half of the enrolled patients received vasoactive agents for septic shock and a similar fraction met criteria for ALI (PaO_2_/FiO_2_ < 300 mmHg) with the majority having its more severe form ARDS (PaO_2_/FiO_2_ ≤ 200 mmHg). In the development cohort, the 2009 pandemic H1NI was the most common strain identified, whereas influenza A H3N2 was commonly identified in the validation cohort.

**Table 1:** Characteristics of children admitted to the pediatric with influenza-associated critical illness in the development and validation cohorts.

Demographics and PICU course	Development (*N* = 171)	Validation (*N* = 76)	*p*-Value
Age (years), median [interquartile range (IQR)]	6.5 (3.4, 10.9)	6.6 (1.8, 11.8)	0.81
Male, *N* (%)	102 (59.6)	38 (50.0)	0.20
Hispanic, *N* (%)	42 (24.6)	19 (25.0)	0.94
Race, *N* (%)			
White	128 (74.8)	55 (72.4)	0.80
African-American	21 (12.3)	15 (19.7)	0.18
Other	22 (12.9)	6 (7.9)	0.36
No influenza risk conditions	105 (61.4)	50 (65.8)	0.61
Mechanical ventilation			
None	15 (8.8)	7 (9.2)	0.91
Non-invasive only	22 (12.9)	10 (13.2)	0.95
Any invasive	134 (78.4)	59 (77.6)	0.90
Acute lung injury/ARDS	81 (47.4)	30 (39.5)	0.31
PaO_2_/FiO_2_, median (IQR)			
Shock	79 (46.2)	38 (50)	0.68
Extracorporeal life support	23 (13.5)	11 (14.5)	0.99
Days in PICU, median (IQR)	7.3 (4, 13.8)	6.1 (2.8, 12.9)	0.92
Hospital mortality	11 (6.4)	2 (2.6)	0.36
Pathogens identified, *N* (%)			
Influenza infections			
Influenza A	140 (81.9)	59 (77.6)	
2009 H1N1	84 (49.1)	0^a^	
H3N2	26 (15.2)	28 (36.8)	**0.0003**
Other or no subtype	30 (21.4)	29 (38.2)	
Influenza B	32 (18.7)^a^	17 (22.4)	0.61
Bacterial pathogen identified	65 (38.0)	24 (31.6)	0.41
Non-influenza viral pathogen identified	19 (11.1)	21 (27.6)	**0.002**

*^a^Some with no subtype could be 2009 H1N1; one patient positive for influenza A 2009 H1 and influenza. Bold means p-values were less than 0.05*.

In the development cohort, there were 70 (40.9%) with no shock or ALI, 22 with ALI only (12.9%), 20 with shock only (11.7%), and 59 with both (34.5%). In children with both ALI and shock, 32% received ECMO and 17% died versus 0 and 1% of children without either ALI or shock; survivors of both also had significantly longer PICU length of stay. Children with ALI and shock were older [median age 9.4 years, interquartile range (IQR) 5.7, 13.8]. Influenza B and bacterial superinfection with methicillin-resistant *S. aureus* were also more prevalent in the children with both ALI and shock (30.5 and 37.3%, respectively). Detailed comparisons of demographic characteristics, clinical outcomes, and infections for these outcome subgroups are shown in Tables S1 and S2 in Supplementary Material.

Serum samples from the BS were provided at enrollment (mostly within 24 h) by 165 of 171 influenza-positive children in the development cohort, with endotracheal samples (ET) available from 93 patients on mechanical ventilator support (87 of 93 also had a matched BS sample). We measured the concentrations of 42 cytokines, chemokines, and other immune signaling analytes (referred to hereafter as cytokines) in BS and ET samples (Figure S1 in Supplementary Material).

### Strong Cytokine Covariation Motivated Use of Relative Cytokine Concentrations

**Figure 1:**
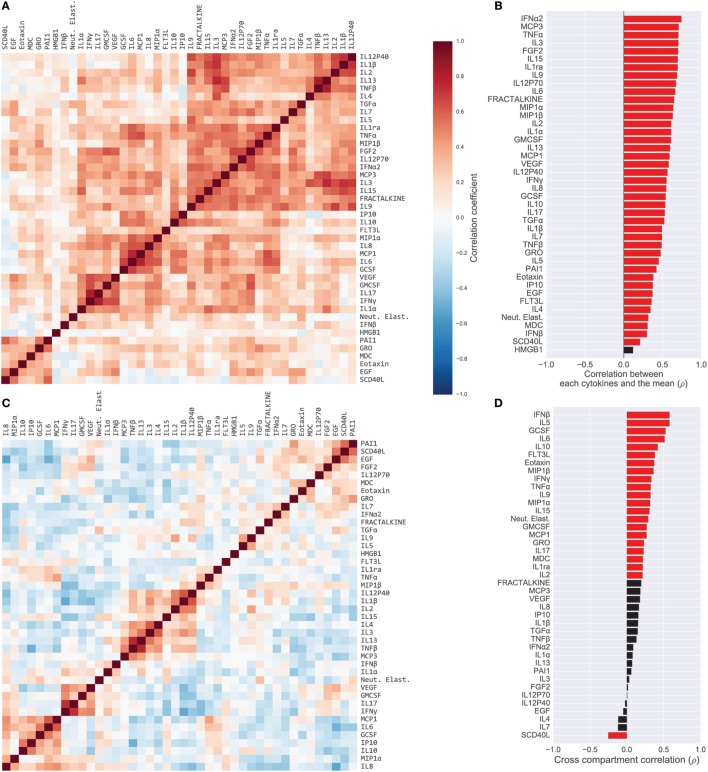
Cytokine covariation. **(A)** Pairwise Pearson’s correlations among log-concentrations of cytokines, chemokines, and growth factors in serum samples (165 patients; 42 cytokines). Cytokines were sorted along both axes to emphasize clusters of cytokines, using hierarchical clustering (complete-linkage). **(B)** Correlation of cytokines with each patient’s mean cytokine concentrations. Red bars indicate correlation with unadjusted *p* < 0.05. **(C)** Pairwise correlations of cytokines after computing relative concentrations adjusted for each patient’s mean cytokine concentration. **(D)** Correlation of each cytokine in serum versus endotracheal aspirate samples. Red bars indicate correlation with unadjusted *p* < 0.05.

Most cytokines in BS samples were positively correlated. We quantified this by computing the Pearson’s correlation coefficient between all pairs of BS cytokines (Figure 1A). Evidenced by the majority of red-shade areas of the heatmap in Figure 1A we noted that 70% of all pairs of cytokines exhibited a positive correlation (unadjusted *p* < 0.05) with a median correlation coefficient of 0.26 [IQR (0.13, 0.39)]. Correlations were also high among ET cytokines with a median correlation coefficient of 0.37 [IQR (0.23, 0.52); Figure S2A in Supplementary Material]. Therefore, within a compartment, children with a high concentration of one cytokine were relatively likely to have high concentrations of all other cytokines. With the exception of one BS cytokine and one ET cytokine, the levels of all cytokines were correlated with the mean cytokine level within each compartment (*p* < 0.05 unadjusted; Figure 1B for BS; Figure S2B in Supplementary Material for ET). In contrast, as evidenced by the majority of blue-shaded regions in Figure 1C, correlations between individual cytokines measured in the BS with the same cytokine in the ET compartment [median 0.21, IQR (0.08, 0.32)] were similar to or weaker than the within-compartment correlation of all measured BS or ET cytokines. A Wilcoxon rank-sum test comparing the intercompartment correlation coefficients to the within-compartment coefficients for BS cytokines showed that the difference was significant (*p* < 0.04); the difference was also significant for intercompartment correlations and ET cytokines (*p* < 0.001). For example, the correlation coefficient of IP10 measured in BS and ET (i.e., intercompartment correlation) was 0.15 while the median correlation of ET IP10 with all other ET cytokines was 0.27 [IQR (0.15, 0.41)] (Figure S3 in Supplementary Material).

We hypothesized that the high levels of intrasample correlation reflected generalized inflammation or possibly sampling artifacts (e.g., ET sample dilution), both of which could obscure important immunological signals. Therefore, from the absolute cytokine concentrations we derived relative concentrations that compensated for the mean concentration of cytokines in each sample; a high relative concentration reflected that the cytokine was high *relative* to the all of the other cytokines in the sample (see Materials and Methods for details). The relative concentrations revealed correlations that were quite different from those computed with absolute concentrations (Figure 1C for BS; Figure S4 in Supplementary Material for ET). For example, unadjusted BS levels of IL9 and TNFα were positively correlated, both with each other (ρ = 0.37) and with the compartment mean (ρ = 0.68 and ρ = 0.70, respectively), yet the relative concentrations were negatively correlated (ρ = −0.27). The change in correlation reflects that patients with generally high cytokine levels tended to have high IL9 and high TNFα, yet after accounting for overall levels of inflammation, the patients with relatively high IL9 were likely to have *relatively* lower TNFα. Many correlations were salient in both the absolute and relative cytokine datasets, for example, IL6 and IL8, which were strongly correlated in the blood (absolute, ρ = 0.63; relative, ρ = 0.43). Overall, the percentage of significantly correlated cytokine pairs (unadjusted *p* < 0.05) dropped from 70 to 42% in the blood and from 79 to 38% in the lung after adjusting for mean cytokine concentration.

### Cytokine Modules Associated With Illness Severity

**Figure 2:**
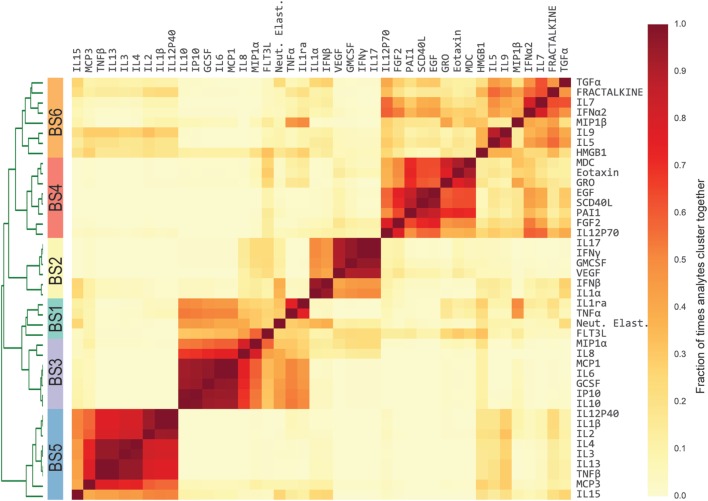
Modules of blood stream (BS) cytokines based on relative cytokine concentrations. Heatmap of BS cytokine modules. A distance was computed between every pair of cytokines using Pearson’s correlation coefficient estimated across the development cohort. Cytokines were then clustered using complete-linkage hierarchical clustering. The algorithm was repeated 1,000 times, each time resampling the patient cohort with replacement [i.e., patient-level bootstrap clustering (25)]. Finally, cytokines were clustered based on the fraction of times that each pair of cytokines clustered together (heatmap color intensity). Dendrogram shows the separation between clusters, which is the basis of the modules. Stripe of colors indicates the six resultant BS modules used in subsequent analyses.

**Figure 3:**
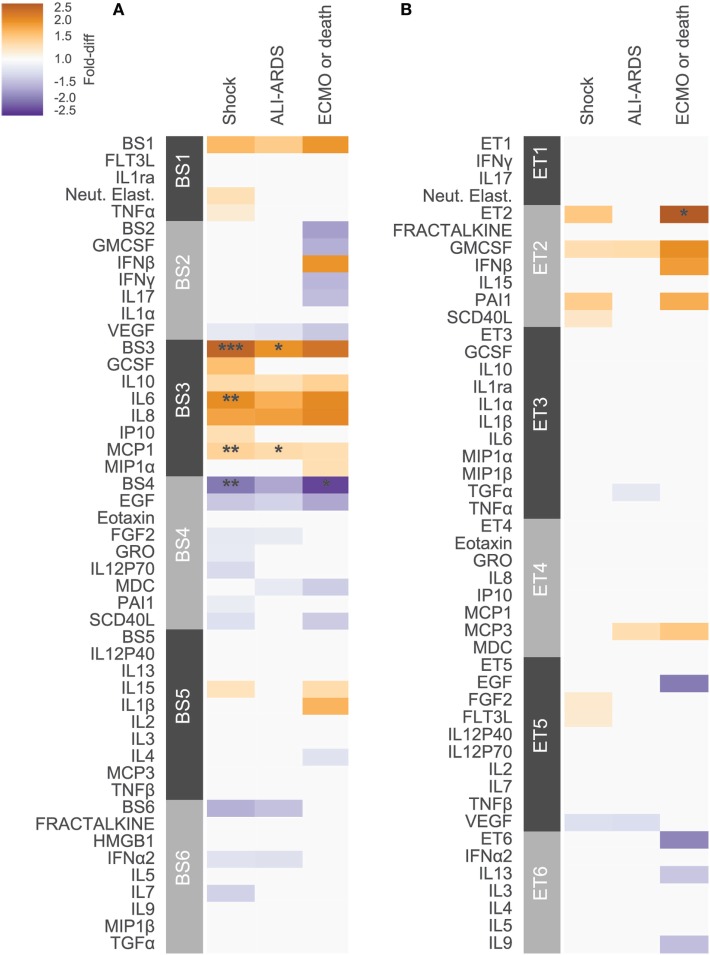
Cytokine associations with clinical complications. Modules constructed of covarying cytokines from **(A)** blood stream (BS) or **(B)** ET samples, were tested for associations with the clinical complications shock, acute lung injury (ALI)-ARDS and ECMO-death. Each cytokine or module is indicated along the rows, grouped by their assigned module. Heatmap color indicates the direction and magnitude of the fold-difference between patients with and without the complication in the development cohort (*N* = 165). Only associations with false-discovery rate (FDR)-adjusted *q*-value < 0.2 are colored. Asterisks indicate family-wise error rate (FWER)-adjusted *p*-values with ***, **, and * indicating *p* < 0.0005, 0.005, and 0.05, respectively.

**Figure 4:**
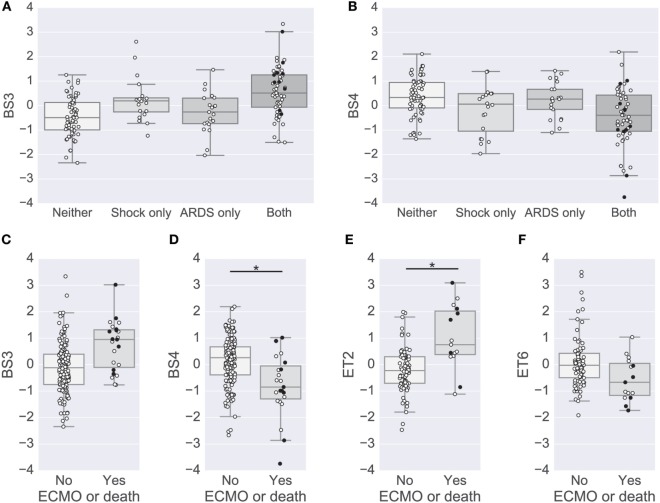
Modules associated with clinical complications. **(A,B)** Box plots of the BS3 and BS4 cytokine module levels for patients with or without shock and acute lung injury (ALI)-ARDS. BS3 levels were significantly different in patients with versus without shock and patients with versus without ALI-ARDS [family-wise error rate (FWER)-adjusted *p*-values < 0.01]. BS4 levels were significantly different in patients with versus without shock (FWER-adjusted *p*-value < 0.01). **(C–F)** Box plots of cytokine module levels in patients for the BS3 **(C)**, BS4 **(D)**, ET2 **(E)**, and ET6 **(F)** modules grouped by whether or not they died or were on ECMO support (near death). Extents of the box indicate the interquartile range (IQR) with whiskers indicating the most extreme data point within 1.5 times the IQR. Patients who died on study are plotted using a black circle. An asterisk and line indicate a FWER-adjusted *p*-values < 0.05 **(D,E)**.

Previous studies have conducted univariate analyses of cytokines to assess phenotypic associations. This approach has limited statistical power and does not leverage the complex covariation structure. We applied a modular approach that clusters cytokines, based on their pairwise correlation, to both amplify the signal they share and aid in interpretation by grouping putatively cosignaling molecules (see Materials and Methods for details). Cytokines in BS and ET samples were clustered independently due to the low cross-compartment correlations, which reflect notable differences in signaling patterns (Figure 1D). The approach yielded six BS (Figure 2) and six ET (Figure S5 in Supplementary Material) modules that were based on pairwise correlations of relative cytokine concentrations. A patient’s score for each module was based on the mean cytokine concentration within the module. We then conducted a prespecified primary analysis testing BS and ET modules for associations with clinical complications: (1) septic shock, (2) ALI/ARDS, (3) mechanical ventilation, and (4) ECMO support or death (ECMO-death). We identified several significant associations with BS and ET modules (Figure 3; Tables S3 and S4 in Supplementary Material), each of which was determined to be independent of bacterial coinfection and age. The BS3 module containing GCSF, IL6, IL8, IL10, IP10, MCP1, and MIP1α was positively associated with shock (OR 3.37, FWER *p* < 10^−4^) and ALI/ARDS (OR 2.15, FWER *p* = 0.008), with a trend for association with ECMO-death (OR 2.33, FWER *p* = 0.085; Figure 4). An examination of individual cytokines within BS3 showed that patients with shock had higher MCP1 (1.4-fold), IL6 (2.0-fold), GCSF (1.6-fold), and IL8 (1.8-fold), however, only IL6 and MCP1 were significantly associated with shock and ALI/ARDS as individual cytokines after FWER-adjustment (Table S5 in Supplementary Material). A second module, BS4, was inversely associated with shock (OR 0.43, FWER *p* = 0.002) and ECMO-death (OR 0.43, FWER *p* = 0.04); all cytokines were lower in patients with septic shock, including EGF, GRO, IL12-P70, sCD40L, eotaxin, FGF2, and PAI-1. Notably, even the most strongly associated cytokine, EGF (OR 0.11), was not significantly associated after FWER-adjustment, demonstrating the increased sensitivity of a modular approach. A module of endotracheal cytokines, ET2, was significantly associated with ECMO-death (OR 3.6, FWER *p* = 0.03; Figure 4E). All cytokines within the module were higher in patients who died or who received ECMO, including GMCSF (2-fold), IFNβ (1.9-fold), and PAI-1 (1.7-fold), but were not significantly associated with ECMO-death when analyzed individually (Table S6 in Supplementary Material).

**Figure 5:**
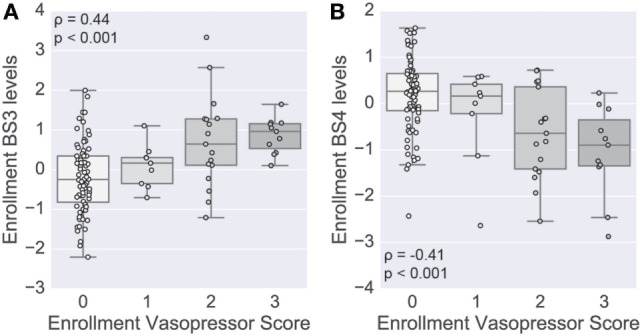
Shock-associated serum modules correlate with shock severity. A score based on vasopressor levels was computed at enrollment in a subset of patients (*n* = 85). Boxplots show the distribution of the levels of the BS3 **(A)** and BS4 **(B)** cytokine modules at enrollment (log scale). Module levels are grouped by each patient’s cardiovascular sepsis-related organ failure assessment (CV-SOFA) score at enrollment. Spearman’s rank-based correlation was used to assess correlation between the vasopressor score and cytokine module levels, with the correlation coefficient and *p*-value annotating each panel.

To complement the primary analysis of septic shock, we additionally quantified the severity of shock based on the level of vasopressors administered captured by the modified CV-SOFA score, which is used clinically to track a patient’s severity (21). We found that patients’ levels of BS3 and BS4 cytokines were significantly positively and negatively correlated, respectively, with their CV-SOFA scores at enrollment (*n* = 85; BS3 Spearman’s ρ = 0.44, *p* < 0.001, Figure 5A; BS4 ρ = −0.41, *p* < 0.001, Figure 5B).

Analyses were conducted in parallel using absolute cytokine concentrations and their modules (denoted AbsBSX). Twelve of 42 cytokines (28.6%) were in the same BS modules and 16 of 42 (38.1%) were in the same ET modules (Figure S6 in Supplementary Material). The cytokines in BS3 were identical to those in AbsBS3 and the module was also positively associated with shock (OR 2.5, FWER *p* = 0.002) and ECMO-death (OR 2.6, FWER *p* = 0.002); it did not reach significance for ALI/ARDS (OR 1.9, FWER *p* = 0.1) (Tables S7 and S8 in Supplementary Material). AbsBS1 containing IFNβ, FLT3L, neutrophil elastase, and HMGB1 was also positively associated with ECMO-death (OR 2.98, FWER *p* = 0.01). No AbsBS or AbET modules were inversely associated with clinical outcomes. No absolute individual BS or ET cytokines or ET cytokine modules were significantly associated with outcomes after multiplicity adjustment (Tables S9 and S10 in Supplementary Material; see Presentation S1 in Supplementary Material for additional comparisons of relative and absolute modules).

### Independent Validation of Significant Associations

In the validation cohort, we evaluated each of the significant associations that were identified between the BS3 and BS4 cytokine modules and clinical complications. Despite the smaller cohort, all findings were replicated (Table S11 in Supplementary Material), including both the direct associations of BS3 with ALI, shock, and ECMO-death (all *p* < 0.001) as well as the inverse associations of BS4 with shock (*p* = 0.04) and ECMO-death (*p* = 0.0003). We also found that additional associations in the development cohort with FDR-*q* < 0.2 were replicated in the validation cohort. There were insufficient ET validation samples to validate ET module results.

### Blood Cytokines Can Be Used As Biomarkers of Clinical Severity

**Figure 6:**
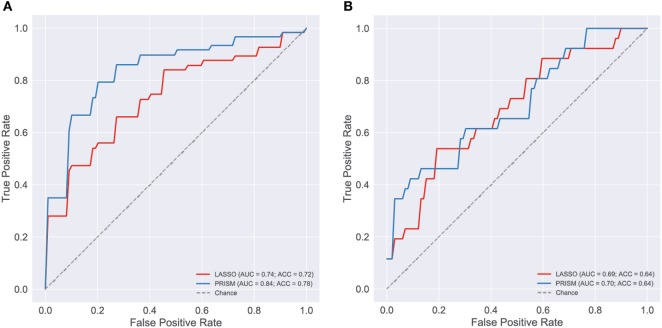
Evaluation of blood cytokine classifier. To evaluate blood stream (BS) cytokines as a potential biomarker of the most severely ill patients [shock and acute lung injury (ALI)/ARDS] a classifier was developed using L1-regularized logistic regression (i.e., LASSO). The final classifier was based on age and the concentrations of 19 cytokines: TNFα, IFNα2, GMCSF, GRO, IL1β, IL6, IL7, IL8, IL10, MCP1, MCP3, MDC, MIP1β, VEGF, IFNβ, EGF, FGF2, TGFα, and HMGB1. For reference, a classifier based on pediatric risk of mortality (PRISM) score and age alone was also evaluated. Classification performance was evaluated using receiver operating curves (ROC), first on the development cohort [**(A)**, *n* = 165] and then on the validation cohort [**(B)**, *n* = 73]. The area under the ROC curve (AUC) and classification accuracy (ACC) are provided in each panel (see Presentation S1 in Supplementary Material for classifier implementation and evaluation details).

To provide more rapid and appropriate treatment to children in the PICU, there is a need to identify the most severely ill patients: those with both septic shock and ALI/ARDS. We hypothesized that a biomarker based on cytokines in the blood could provide useful diagnostic information. We applied three common machine-learning algorithms to the cytokine data from the development cohort and evaluated their ability to discriminate patients with shock and ALI/ARDS from the others: (1) logistic regression with L1-regularization (LASSO), (2) support vector machine classifier with L2-regularization (SVC), and (3) gradient boosting machine classifier. Both relative and absolute cytokine concentrations were considered in two independent analyses. Classification performance was evaluated in 10-fold nested cross-validation using the area under the receiver operator curve (AUC), sensitivity (i.e., true-positive rate), and specificity (i.e., one minus the false-positive rate). The classifiers based on absolute cytokine concentration performed well (Figure 6; Table 2), with general concordance among the algorithms; all had AUC between 0.74 and 0.78 and there was high correlation in the predicted probability of each patient’s phenotype (Spearman ρ = 0.8–0.92 for each pair of classifiers). The LASSO classifier had the additional practical advantage of finding a sparse solution requiring only the concentrations of 19 of the 42 cytokines, including many that were implicated in the primary analysis: TNFα, IFNα2, GMCSF, GRO, IL1β, IL6, IL7, IL8, IL10, MCP1, MCP3, MDC, MIP1β, VEGF, IFNβ, EGF, FGF2, TGFα, and HMGB1. Use of relative cytokine concentrations did not improve performance with AUC ranging from 0.70 to 0.74 (Table S12 in Supplementary Material). After training the classifiers on patients in the development cohort, they were used to predict the severity phenotype of patients in the validation cohort. Performance declined in the validation cohort with AUCs ranging from 0.69 to 0.71.

**Table 2:** Biomarker development with absolute analyte concentrations.

	Development			Validation		
	Area under the receiver operator curve (AUC)	Sensitivity	Specificity	AUC	Sensitivity	Specificity
LASSO	0.74	0.51	0.82	0.69	0.54	0.70
SVC	0.78	0.53	0.90	0.71	0.54	0.77
Gradient boosting machine classifier	0.74	0.44	0.91	0.70	0.54	0.81
Pediatric risk of mortality	0.84	0.61	0.87	0.70	0.46	0.75

The cytokine-based classifiers were compared to a logistic regression model using each patient’s age and admission day illness severity score (PRISM III score) measuring degree of organ dysfunction based on patient physiology and excluding level of support. The score was developed as a mortality risk assessment tool in the PICU (26). Notably, many of the physiological measures that contribute to the PRISM score are also included in the definition of septic shock and ALI, making it a somewhat circular classifier for the shock and ALI severity phenotype. The model performed well with AUC of 0.84 in the development cohort, however, performance was no better than cytokines on the validation cohort with AUC of 0.7.

## Discussion

Children who develop the most severe complications of influenza infection, including ALI/ARDS and septic shock, are the focus of critical care management and could potentially benefit from targeted immunomodulatory therapy. However, a major hurdle will be to dissect the pathways that can be targeted to reduce pathogenesis while maintaining the ability of host cells to mount an antiviral response (3). Although mouse models of influenza-associated lung injury exist, data describing immune responses in children with respiratory failure and influenza lung injury is lacking. Our central objective was to gain insight into the pathogenesis of lung injury in children with influenza, with the ultimate goal of guiding strategies for future therapies or biomarkers. In two independent cohorts, we identified two distinct modules of serum cytokines associated with shock, ALI and ECMO-death independent of age and bacterial coinfection. One module (BS4) included eotaxin, EGF, FGF2, GRO, IL12P70, PAI-1, and soluble CD40L and was relatively deficient in children with shock and in those who received ECMO or died. Consistent with these findings, the module was negatively correlated with vasopressor levels, an indicator of shock severity.

The inverse association of EGF with severity is consistent with two smaller studies of acute influenza infection, in which EGF was lower in patients that required intensive care (19) or hospitalization (22). EGF plays a role in the regeneration of damaged epithelium (13); inhibition of downstream EGF receptor tyrosine kinase impedes epithelial repair in mice with ALI (27) and alveolar epithelial wound healing depends on EGF signaling (28). Two other growth factors, FGF2 (a BS4 member) and VEGF, were also lower in serum of patients that died. We also noted trends of relatively lower EGF in ET samples of patients that died, representing one of only a few cytokines showing similar associations in the blood and lung. Together these findings indicate that one component of cytokine dysregulation may be a reduction in the ability to stimulate barrier repair necessary for recovery. This motivates further study into stimulation of epithelial repair in the treatment of severe influenza, particularly in patients beyond the acute phase of infection. For example, a recent study in mice reported that bone marrow-derived mesenchymal stromal cell therapy can enhance *in vitro* alveolar human type 2 epithelial function and increase survival in H5N1 influenza infection (29).

A second serum module (BS3), which included IL6, IL8, IL10, IP10, GCSF, and MCP1, was elevated in patients with septic shock, ALI, and ECMO-death, in two independent cohorts. In a previous study of influenza-infected PICU patients, all but GCSF and IL10 were previously associated with mortality (17). Studies of pandemic H1N1, associated with hospitalizations and death especially in children (30), have also reported increases in IL6, IL8, IL10, MCP1, MIP1β, and IP10 compared to cases of seasonal influenza and healthy controls (15, 31–33). Adults infected with avian H5N1 (versus human H3/H1), also had elevated serum concentrations of IP10, MCP1, IL10, and IL6 that were highly correlated with viral load (6). Notably, IL8, IL6, MCP1, and GCSF have been associated with ARDS, mortality and organ dysfunction in critically ill patients without influenza infection (34–36). Therefore, these mediators may not be influenza-specific and may reflect the hyperinflammatory ARDS subphenotype identified by Calfee and colleagues in adult patients (37).

By analyzing cytokines in the blood as well as the lung, at the site of infection, we were able to distinguish unique patterns of immune signaling in the two immune compartments. Cytokines were moderately correlated within compartments and weakly correlated between compartments, consistent with a previous study by our group of a cohort with less severe influenza infection (18). The two compartments differed markedly in their associations with shock and ALI/ARDS, with many cytokines having opposite trends in their associations (Figure S7 in Supplementary Material). The differences may be partially attributed to the lower number of ET samples, but also likely reflect distinct functional responses. Profiling immune signaling in the periphery and at the site of infection may be important in future studies. A limitation of this study is the lack sufficient ET samples in the validation cohort to confirm our findings.

Currently, there is no standard method to adjust absolute cytokine concentrations for sampling effects or for overall cytokine secretion, particularly for respiratory samples that could be influenced by dilution from capillary leak or use of saline. Our novel use of relative cytokine levels, adjusted for the overall mean concentration of all cytokines in a sample, revealed potentially important inverse associations, evidenced by BS4 whose association with shock was not detected using absolute concentrations. There was one module based on absolute concentrations, containing IFNβ, FLT3L, neutrophil elastase, and HMGB1, that was associated with ECMO or death, but did not have an analogous module of relative cytokines with similar associations. Therefore, to fully reveal immune signaling, it may be important to evaluate absolute and relative concentrations; insights from one or the other may differ by context.

Application of a module-based analysis approach appeared overall to increase statistical power, presumably by aggregating covarying signals prior to testing. Although modular analysis has been applied to gene expression studies (38), it has not been applied to multiplexed cytokine protein data. The modules also aided immunological interpretation, generating hypotheses about cytokines that signal together or that share pathways. The combined analysis approach, code for which has been made publicly available, may have broad applicability for multiplexed cytokine analysis in many clinical or scientific contexts.

Modules were not used for development of a cytokine biomarker since interpretability and power for statistical inference were not a requirement. The concordance among three machine-learning algorithms suggested that there was a robust signal in the data. The comparable performance of relative and absolute cytokine concentrations is not surprising as relative concentrations are derived from absolute concentrations. Though the biomarker would need further development and validation before use in the PICU, the LASSO classifier would be most practical as it decreased the number of cytokines required from 42 to 19. The high performance of the PRISM classifier on the development cohort was expected as it is aggregated from several measures of symptom severity, some of which directly inform the diagnosis of shock and ALI. The substantial drop in performance on the validation cohort suggests that the model may be inflexible and overly sensitive at the decision boundary. The validation also showed that a cytokine-based classifier performs comparably to a PRISM-based classifier despite that the latter benefits from direct influence of patient severity. A question for future studies is whether a cytokine-based biomarker could be used earlier, perhaps prior to PICU admission, to identify high risk patients. In this study, we have characterized the state of the immune system in the later stages of infection, when the patient is critically ill. The findings provide new insights into the immune signaling underlying disease pathogenesis and offer potential targets for future investigation.

## Ethics Statement

This study was carried out in accordance with the recommendations of the Institutional Review Boards at each study site with written informed consent from at least one parent or guardian of each subject. The parent or guardian of each subject gave written informed consent in accordance with the Declaration of Helsinki. The protocol was approved by the Institutional Review Boards at each study site.

## Author Contributions

Study design: AR, AP-M, and PALISI. Clinical data collection and management: AA, AP-M, PALISI, and AR. Cytokine data generation: AP-M, AM, and AR. Statistical analysis: AF-G and TH. Synthesis and manuscript writing: AF-G, AP-M, AA, AM, MM, PT, TH, and AR.

## Conflict of Interest Statement

The authors declare that the research was conducted in the absence of any commercial or financial relationships that could be construed as a potential conflict of interest.
