# Supplementary material for: Cytokine Profiles of Severe Influenza Virus-Related Complications in Children
Source: Front Immunol. 2017 Nov 6;8:1423. doi: 10.3389/fimmu.2017.01423 (PMC5681736; doi:10.3389/fimmu.2017.01423)
Supplement: Supplementary file 1 [file presentation_1.pdf]

Supplementary Material

Cytokine profiles of severe influenza virus-related complications in children

A. Fiore-Gartland, A. Panoskaltsis-Mortari, A. A. Agan, A. J. Mistry, P. G. Thomas, M. A. Matthay, PALISI PICFlu Investigators, T. Hertz, A. G. Randolph

Supplemental Methods

Cytokine assays

Patient sera and endotracheal aspirates were analyzed for cytokines, chemokines, growth factors and other mediators at the University of Minnesota Cytokine Reference Laboratory using the Luminex 200 platform (Austin, TX) or by standard ELISA with human-specific reagents. Luminex assays were analyzed with Bioplex 2.0 software (BioRad, Hercules, CA). ELISA plates were read on a BioRad 550 reader with Microplate Manager software. Reagents used, along with sensitivity levels were as follows: A 38-plex (EMD Millipore, Billerica, MA) included sCD40L (5.0 pg/mL), EGF (3.0 pg/mL), Eotaxin (CCL11, 3.2 pg/mL), FGF2 (20 pg/mL), Flt-3L (4.0 pg/mL), Fractalkine (20 pg/mL), G-CSF (6.0 pg/mL), GM-CSF (0.1 pg/mL), GRO (CXCL1, 8.0 pg/mL), IFNa2 (1.8 pg/mL), IFNg (0.4 pg/mL), IL-1a (2.6 pg/mL), IL-1b (0.3 pg/mL), IL-1ra 0.2 pg/mL), IL-2 (0.1 pg/mL), IL-3 (0.1 pg/mL), IL-4 (4.0 pg/mL), IL-5 (1.0 pg/mL), IL-6 (0.1 pg/mL), IL-7 (0.4 pg/mL), IL-8 (0.2 pg/mL), IL-9 (0.7 pg/mL), IL-10 (0.5 pg/mL), IL-12p40 (3.5 pg/mL), IL-12p70 (2.0 pg/mL), IL-13 (0.8 pg/mL), IL-15 (0.3 pg/mL), IL-17A (0.2 pg/mL), IP-10 (CXCL10, 2.0 pg/mL), MCP-1 (CCL2, 2.0 pg/mL), MCP-3 (CCL7, 8.0 pg/mL), MDC (CCL22, 2.0 pg/mL), MIP-1a (CCL3, 1.0 pg/mL), MIP-1b (CCL4, 0.5 pg/mL), TGFa (.01 pg/mL), TNFa (0.3 pg/mL), TNFb (0.7 pg/mL), VEGF (40 pg/mL). PAI-1 (10 pg/mL) was analyzed using a single-plex bead set (R&D Systems). ELA-2 (neutrophil elastase, 10 ng/mL) was analyzed with a single-plex bead set (EMD Millipore). HMGB1 (0.1 ng/mL) was measured by ELISA (Antibodies-online, Atlanta, GA). IFNb was measured by ELISA (PBL). Total protein levels in endotracheal aspirates were determined by the Bradford method (reagent from Sigma, St. Louis, MO). All samples were run in duplicate, sample volume permitting. Replicates with a coefficient of variation > 10% were considered for reanalysis, volume permitting. All values were interpolated from standard curves of the corresponding human recombinant proteins run on every plate. Measurements that were below the level of detection (LOD) were assigned the LOD specified by the manufacturer for each analyte. Sensitivity analyses of analyte correlation and clustering were performed with LOD values assigned LOD/sqrt(2); no substantial differences were noted, partly due to the low number of samples with concentrations below LOD.

Modular cytokine analysis

Cytokine concentrations (pg/mL) were log-transformed for all analyses. Cytokine levels were highly correlated across participants. To adjust for this, measurements of each cytokine were regressed on a vector of the mean cytokine levels for each participant. The residuals of each regression were used in subsequent analyses as a "relative" cytokine concentration (i.e. relative to the mean). Formally, the Pearson's correlation coefficient between two relative cytokine concentrations is the partial correlation coefficient of the two, adjusted for the mean cytokine level. The relative and absolute individual cytokine concentrations and cytokine modules were analyzed in parallel using identical methods, with multiplicity adjustment across all tests including those involving relative and absolute variables.

Cytokine modules were formed based on the correlation of relative and absolute cytokine levels in ET and BS samples (four sets of modules in total). Complete-linkage agglomerative hierarchical clustering was used to cluster the variables with the Pearson's correlation coefficient as the similarity metric. Complete linkage, which joins subclusters iteratively based on the maximum distance between pairs of variables in the subclusters, was used because it tends to form compact clusters. Since Pearson's correlation is mean and scale invariant, no further transformations were necessary to perform clustering.

The number of clusters (K) was fixed for all cluster analyses to facilitate comparisons and was determined using the Tibshirani "gap statistic" (1), which analyzes the marginal decreases in intra-cluster distance (ICD) with increases in K from 1 to 20 using a null permuted dataset as a reference. The ICD decreases with every increase in K, however, the "ideal"/natural number of clusters according to Tibshirani, is the smallest K for which all decreases in ICD are greater than for the null/permuted dataset. By using a data-driven approach to determining K we have standardized this type of analysis across cytokine datasets in multiple contexts that may have different natural values for K. Though the number of clusters ranged between 5 and 8 for ET/BS and relative/absolute datasets we chose to use 6 clusters for all analyses, to ease comparison of clusters. Furthermore, the arbitrary names of the clusters in each dataset were renamed to maximize the overlap between clusters in the BS/ET and relative/absolute sets of clusters.

To increase the stability of clusters we employed bootstrap clustering methods that are common in gene expression microarray analysis (2). We repeated the clustering procedure described above on participant-bootstrapped datasets 1000 times. Over the iterations, we recorded the number of times that each pair of cytokines clustered together. Conceptually this can be thought of as a bootstrap estimate of cluster membership, simulating the reliability of the clusters in repeated experiments under the same conditions. We performed the final hierarchical cluster analysis on this matrix of reliability fractions. Module scores were computed as the mean of the members of each cluster after standardizing each to mean zero and unit variance.

The Primary Analysis of cytokine modules included tests for associations with four clinical measures of disease severity: (1) septic shock requiring vasopressors, (2) ALI or ARDS, (3) mechanical ventilation and (4) ECMO support or death (ECMO-death). With a goal of controlling for multiple comparisons without missing important signals, the Primary Analysis tested for an association of each module with each of the clinical outcomes listed above, subject to control of the family-wise error rate (FWER) at 5%. This was followed by the Exploratory Analysis, which tested individual cytokines subject to control of the false discovery rate (FDR) at 20%. In both the Primary and Exploratory analyses, the modules or cytokines were tested in univariate models, and in multivariate models adjusting for bacterial coinfection (BCo) and age, on the basis that a substantial fraction of patients presented with BCo and BCo is a known complication associated with clinical severity. In the Primary Analysis we performed 182 total tests that were subject to multiplicity adjustment. This included 96 tests of BS modules (6 modules, 4 clinical variables, relative and absolute data, adjusted and unadjusted for BCo and age), 72 tests of ET modules (6 modules, 3 clinical variables, relative and absolute data, adjusted and unadjusted for BCo and age) and 14 tests with the BS and ET mean cytokine concentrations (4/3 clinical variables, adjusted and unadjusted for BCo and age). ET modules were not tested for association with ventilation since all patients who provided an ET sample were ventilated.

Python code for all statistical analyses is publicly available on github: https://github.com/agartland/cycluster

Biomarker development

We applied three common machine learning algorithms to the cytokine data: (1) logistic regression with L1-regularization (LASSO), (2) support vector machine classifier with L2-regularization (SVC), (3) gradient boosting machine classifier (GBMC). Each model had hyper-parameters that were tuned in nested 10-fold cross-validation (CV): (1) LASSO regularization (*C*), (2) SVC regularization (*C*) and the radial basis function (RBF) kernel coefficient (*gamma*) and (3) GBMC learning rate and maximum depth. Classification performance was evaluated using the outer 10-fold cross-validation, so that hyper-parameters were tuned on different data than what was used for evaluation. Hyper-parameters were tuned on inner CV training data, minimizing a logistic loss function. The AUC, sensitivity, specificity and accuracy (ACC) were reported as the mean across the 10 test folds. A “final” model was fit using all the data and optimal hyper-parameters, for classifying patients in the validation cohort. The final GBMC and SVC models required all cytokines whereas the LASSO model found a sparse solution that only required 19 cytokines and age due to the L1-regularization: TNFα, IFNα2, GMCSF, GRO, IL1ß, IL6, IL7, IL8, IL10, MCP1, MCP3, MDC, MIP1ß, VEGF, IFNß, EGF, FGF2, TGFα, HMGB1. We considered both absolute (**Table 2**) and relative (**Table S12**) cytokine concentrations as two independent datasets. All models also included age as a predictor. In the development dataset each cytokine variable was standardized to have mean zero and standard deviation one; the identical standardization was applied to each variable in the validation dataset (i.e. using the sample mean and standard deviation from the development dataset). Missing values were imputed using the mean concentration for each cytokine and only for the purposes of biomarker development (not the primary analysis). Classification was performed using python and the open-source scikit-learn machine learning package (3), with code available upon request.

Patient enrollment statistics by site

Patients from the development and validation cohort were enrolled at the following 35 sites: Akron Children's Hospital, Akron, OH; Arkansas Children’s Hospital, Little Rock, AR; Banner Children’s/Diamond Children’s Medical Center, Tucson, AZ; Boston Children’s Hospital, Boston, MA; Centre Hospitalier de l’Université Laval, Quebec , Quebec, Canada.; Children’s Healthcare of Atlanta at Egleston, Atlanta, GA; Children’s Hospital at Dartmouth-Hitchcock, Dartmouth, NH; Children’s Hospital Central California, Madera, CA; Children’s Hospital Colorado, Aurora, CO; Children’s Hospital Los Angeles, Los Angeles, CA; Children’s Hospital of Nebraska, Omaha, NE; Children’s Hospital of Orange County, Orange, CA; Children’s Hospital of Philadelphia, Philadelphia, PA; Children’s Hospital of Wisconsin, Milwaukee, Wisconsin; Children’s Hospitals and Clinics of Minnesota, Minneapolis, MN; Children’s Medical Center of Dallas, Dallas, TX; Connecticut Children’s Medical Center, Hartford, CT; Dell Children’s Medical Center of Central Texas, Austin, TX; Golisano Children’s Hospital, Rochester, NY; Holtz Children’s Hospital, Miami, FL; Johns Hopkins Children’s Center, Baltimore, MD; Monroe Carell Jr. Children’s Hospital at Vanderbilt, Nashville, TN; Nationwide Children’s Hospital, Columbus, OH; Norton Children's Hospital, Louisville, KY; Penn State Children’s Hospital, Hershey, PA; Phoenix Children’s Hospital, Phoenix, AZ; Rainbow Babies and Children's Hospital, Cleveland, OH; St. Louis Children’s Hospital, St. Louis, MO; Texas Children’s Hospital, Houston, TX; The Children’s Hospital at Montefiore, Bronx, NY; The University of Chicago Medicine Comer Children’s Hospital, Chicago, IL; UCSF Benioff Children’s Hospital Oakland, Oakland, CA; UCSF Benioff Children’s Hospital, San Francisco, CA; University of Virginia Children’s Hospital, Charlottesville, VA; Yale-New Haven Children's Hospital, New Haven, CT. The distribution of patients enrolled at these sites is described in **Table S13**.

Supplementary Results

Modules based on absolute cytokine concentrations were weakly or not significantly associated with illness severity

Though we hypothesized that relative cytokine levels would lead to more immunologically relevant modules, we also conducted a parallel set of analyses using absolute cytokine concentrations to form modules and test for associations with illness severity. We also applied the module formation algorithm to absolute cytokine concentrations. Only the absolute BS3 module (AbsBS3) included the same seven cytokines; all other BS and ET modules were different, although there was some similarity in the groupings of cytokines (**Figure S6**). We quantified the similarity of relative and absolute modules using a mutual information statistic that is adjusted for module member overlap by chance (0 = no significant overlap to 1 = identical). Relative and absolute modules had higher similarity in the blood (AMI = 0.58) than in the lung (AMI = 0.4).

The absolute modules were tested for associations with illness severity, with multiplicity adjustment applied across all tests involving relative and absolute modules. For the BS3 module, the absolute cytokines were similarly associated with shock, ALI/ARDS and ECMO-death, though the association with shock was less salient (OR 2.5 vs. 3.4) (**Table S7**). The AbsBS1 module, which included IFNβ, FLT3L, neutrophil elastase and HMGB1 was also associated with ECMO-death (OR 2.98, FWER-p = 0.01). No additional BS or ET absolute modules were significantly associated with illness severity after FWER adjustment (**Table S8**). Notably, though the AbsBS4 module contained many of the same cytokines as the relative BS4 module (AbsBS4 missing FGF2 and IL12-P70), it was only weakly inversely associated with shock (OR 0.64) and was not statistically significant (FWER-p = 1). Together these results demonstrate the importance of using relative cytokine concentrations prior to module formation and integration with clinical data. This was especially important for identifying inverse correlates of illness severity. Tables of associations with absolute cytokine concentrations are presented for completeness (**Tables S9 – S10**).

Supplementary Tables

Table S1. Characteristics and clinical course of critically ill influenza positive patients

Table S2. Identified bacterial and non-influenza viral pathogens in critically ill influenza positive patients

Table S3. Associations with modules of BS cytokines (relative concentrations)

Table S4. Associations with modules of ET cytokines (relative concentrations)

Table S5. Associations with relative BS cytokine concentrations

Table S6. Associations with relative ET cytokine concentrations

Table S7. Associations with modules of BS cytokines (absolute concentrations)

Table S8. Associations with modules of ET cytokines (absolute concentrations)

Table S9. Associations with absolute BS cytokine concentrations

Table S10. Associations with absolute ET cytokine concentrations

Table S11. Validation of BS modules

Table S12. Biomarker development with relative analyte concentrations

Table S13. Distribution of enrollment by study site

Supplementary Figures

Figure S1. Absolute cytokine concentrations
Concentrations of each cytokine in BS (A) and ET (B) samples from 171 influenza-infected children. Cytokines are plotted on a log scale and sorted by median concentration. Extents of each box indicate the inter-quartile range with whiskers indicating the most extreme data-point within 1.5 times the IQR and dots for outliers.

Figure S2. ET cytokine correlations

(A) Pairwise Pearson's correlations among log-concentrations of cytokines and chemokines in ET samples (93 patients; 42 cytokines) (A). Cytokines are sorted along the axes to emphasize clusters of cytokines, using hierarchical clustering (complete-linkage). (B) Correlation of each cytokine with the mean cytokine concentration across patients.

Figure S3. Cross-compartment cytokine correlation

Heatmap of Pearson correlation coefficients of all pairs of cytokines in patient-matched serum and endotracheal aspirate samples (87 patients; 42 cytokines).

Figure S4. ET relative cytokine concentration correlations

Pairwise Pearson's correlations of relative cytokine concentrations in ET samples (93 patients; 42 cytokines). Cytokines are sorted along the axes to emphasize clusters of cytokines, using hierarchical clustering (complete-linkage).

Figure S5. Modules of ET cytokines based on relative concentrations

Heatmap of ET cytokine modules. Each square is shaded based on the fraction of times that the pair of cytokines clustered together in 1000 bootstrap samples of the patients (2). Dendrogram from complete-linkage hierarchical clustering shows the degree of separation between the clusters that form the basis of the modules. Stripe of colors indicates the six resultant ET modules used in subsequent analyses.

Figure S6. Associations with absolute cytokine concentrations

Modules constructed of covarying absolute concentrations of cytokines from (A) BS or (B) ET samples, were tested for associations with the clinical complications shock, ALI-ARDS and ECMO-death. Each cytokine or module is indicated along the rows, grouped by their assigned module. Heatmap color indicates the direction and magnitude of the fold-difference between patients with and without the complication in the development cohort (N = 165). Only associations with FDR-adjusted q-value < 0.2 are colored. Asterisks indicate FWER-adjusted p-values with ***, ** and * indicating p< 0.0005, 0.005 and 0.05, respectively.

Figure S7. Immune profiles of shock in the blood and lung

Cytokines in the blood (*y*-axis) and lung (*x*-axis) plotted according to the magnitude of their association with shock. The association magnitude is represented as the fold-difference in relative concentrations in patients with and without septic shock. Cytokine labels are shown for absolute fold-differences greater than 1.25 times.

Acknowledgements

We gratefully acknowledge the collaboration of the PALISI PICFlu Study Site Investigators who enrolled patients and made other major contributions to this study

1. Arkansas Children’s Hospital, Little Rock, AR: Ronald C. Sanders, MD, Glenda Hefley, RN, MNsc
2. Phoenix Children’s Hospital, Phoenix, AZ: David Tellez, MD, Courtney Bliss, MS, Aimee Labell, MS, RN, Danielle Liss, BA, Ashley L. Ortiz, BA
3. Banner Children’s/Diamond Children’s Medical Center, Tucson, AZ: Katri Typpo, MD, Jen Deschenes, MPH
4. Children’s Hospital Los Angeles, Los Angeles, CA: Barry Markovitz, MD, Jeff Terry, MBA, Rica Sharon P. Morzov, RN, BSN, CPN
5. Children’s Hospital Central California, Madera, CA: Ana Lia Graciano, MD, Melita Baldwin, BS
6. Children’s Hospital of Orange County, Orange, CA: Nick Anas, MD, Adam Schwarz, MD, Chisom Onwunyi, RN, BSN, MPH, CCRP, Stephanie Osborne, RN, CCRC, Tiffany Patterson, BS, CRC, Ofelia Vargas-Shiraishi, BS, CCRC
7. UCSF Benioff Children’s Hospital, San Francisco, CA: Anil Sapru, MD, Maureen Convery, BS, Victoria Lo, BA
8. UCSF Benioff Children’s Hospital Oakland, Oakland, CA: Heidi Flori, MD, Becky Brumfield, RCP, Julie Simon, RN
9. Children’s Hospital Colorado, Aurora, CO: Angela Czaja, MD, Peter Mourani, MD, Valeri Batara Aymami, RN, MSN-CNS, Susanna Burr, CRC, Megan Brocato, CCRC, Stephanie Huston, BS, RPSGT, CRC, Emily Jewett, Danielle Loyola, RN, BSN, MBA
10. Connecticut Children’s Medical Center, Hartford, CT: Christopher Carroll, MD, MS, Kathleen Sala, MPH, Sherell Thornton-Thompson, CCRP
11. Yale-New Haven Children’s Hospital, New Haven, CT: John S. Giuliano Jr., MD, Joana Tala, MD
12. Holtz Children’s Hospital, Miami, FL: Gwenn McLaughlin, MD
13. Children’s Healthcare of Atlanta at Egleston, Atlanta, GA: Matthew Paden, MD, Keiko Tarquinio, MD, Chee-Chee Manghram, Stephanie Meisner, RN, BSN, CCRP, Cheryl L. Stone, RN
14. University of Chicago Medicine Comer Children’s Hospital, Chicago, IL: Juliane Bubeck Wardenburg, MD, PhD, Andrea DeDent, PhD
15. Kosair Children’s Hospital, Louisville, KY: Vicki Montgomery, MD, FCCM, Janice Sullivan, MD, Tracy Evans, RN, Kara Richardson, RN, Melissa Thomas, RN, BSN, CCRC
16. Boston Children’s Hospital, Boston, MA: Adrienne G. Randolph, MD, MSc, Anna A. Agan, BA, Anushay J. Mistry, BS, Ryan M. Sullivan, RN, BSN, CCRN, Stephanie Cobb, BA);

17. Johns Hopkins Children's Center, Baltimore, MD: Melania Bembea, MD, MPH, Elizabeth D. White, RN, CCRP
18. Children's Hospitals and Clinics of Minnesota, Minneapolis, MN: Stephen Kurachek, MD, Angela A. Doucette, CCRP, Erin Olson, RN, CCRP
19. St. Louis Children's Hospital, St. Louis, MO: Mary Hartman, MD, Rachel Jacobs, BA);
20. Children's Hospital of Nebraska, Omaha, NE: Edward Truemper, MD, Machelle Dawson, RN, BSN, MEd, CCRC
21. Children's Hospital at Dartmouth-Hitchcock, Dartmouth, NH: Sholeen Nett, MD, Daniel L. Levin, MD, J. Dean Jarvis, MBA, BSN
22. Children's Hospital at Montefiore, Bronx, NY: Chhavi Katyal, MD
23. Golisano Children's Hospital, Rochester, NY: Kate Ackerman, MD, L. Eugene Daugherty, MD, Laurel Baglia, PhD
24. Nationwide Children's Hospital, Columbus, OH: Mark W. Hall, MD, Kristin Greathouse, BSN, MS , Lisa Steele, RN, BSN, CCRN
25. Penn State Children's Hospital, Hershey, PA: Neal Thomas, MD, Jill Raymond, RN, MSN, Debra Spear, RN
26. Children's Hospital of Philadelphia, Philadelphia, PA: Julie Fitzgerald, MD, Mark Helfaer, MD, Scott Weiss, MD, Jenny L. Bush, RNC, BSN, Mary Ann Diliberto, RN, Brooke B. Park, RN, BSN, Martha Sisko, RN, BSN, CCRC
27. Monroe Carell Jr. Children's Hospital at Vanderbilt, Nashville, TN: Frederick E. Barr, MD
28. Dell Children's Medical Center of Central Texas, Austin, TX: Renee Higgerson, MD, LeeAnn Christie, RN
29. Children's Medical Center of Dallas, Dallas, TX: Cindy Darnell, MD, Shanda Johnson, RRT, MHA, CCRP
30. Texas Children's Hospital, Houston, TX: Laura L. Loftis, MD, Nancy Jaimon, RN, MSN-Ed, Ursula Kyle, MS
31. Children's Hospital of Wisconsin, Milwaukee, WI: Rainer Gedeit, MD, Briana E. Horn, Kate Luther, MPH, Kathy Murkowski, RRT, CCRC
32. University of Virginia Children's Medical Center, Charlottesville, VA: Douglas F. Willson, MD, Robin L. Kelly, RN
33. Centre Hospitalier Universitaire Sainte-Justine, Montreal, Quebec, Canada: Philippe A. Jouvet, MD, Anne-Marie Fontaine, BSc
34. Centre Hospitalier de l'Université Laval, Quebec, Quebec, Canada: Marc-André Dugas, MD

References

1. Tibshirani R, Walther G, Hastie T. Estimating the number of clusters in a data set via the gap statistic. *J. R. Stat. Soc. Ser. B (Statistical Methodol.* [published online ahead of print: 2001]; doi:10.1111/1467-9868.00293

2. Dudoit S, Fridlyand J. Bagging to improve the accuracy of a clustering procedure. *Bioinformatics* 2003;19(9):1090–1099.

3. Pedregosa F et al. Scikit-learn: Machine Learning in Python [Internet]. … *Mach. Learn.* … 2012;12:2825–2830.

Figure S1A. BS absolute analyte concentrations

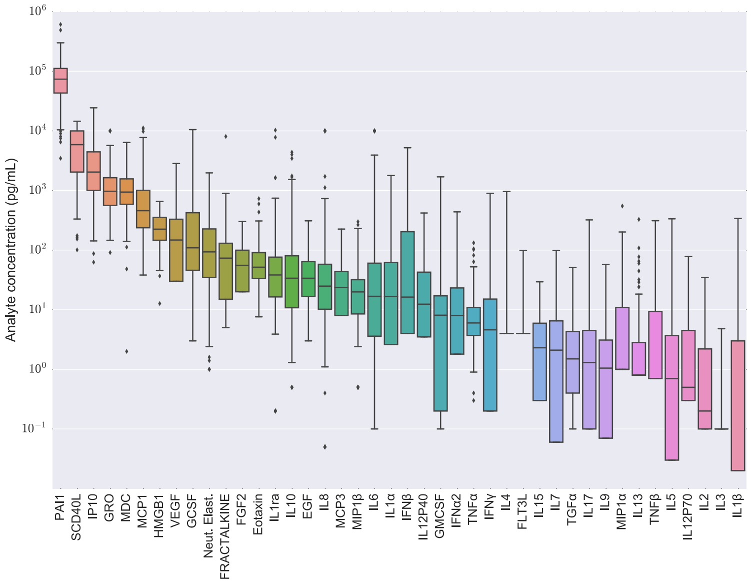

Figure S1B. ET absolute analyte concentrations

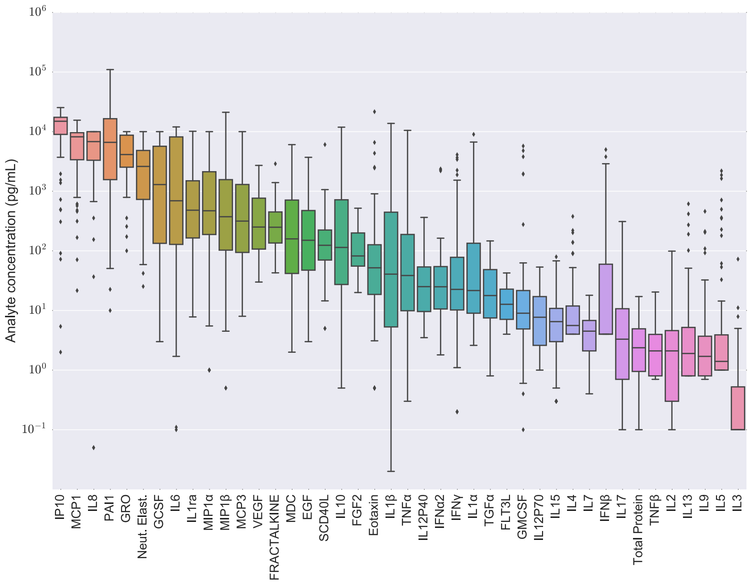

Figure S2A. ET cytokine correlation

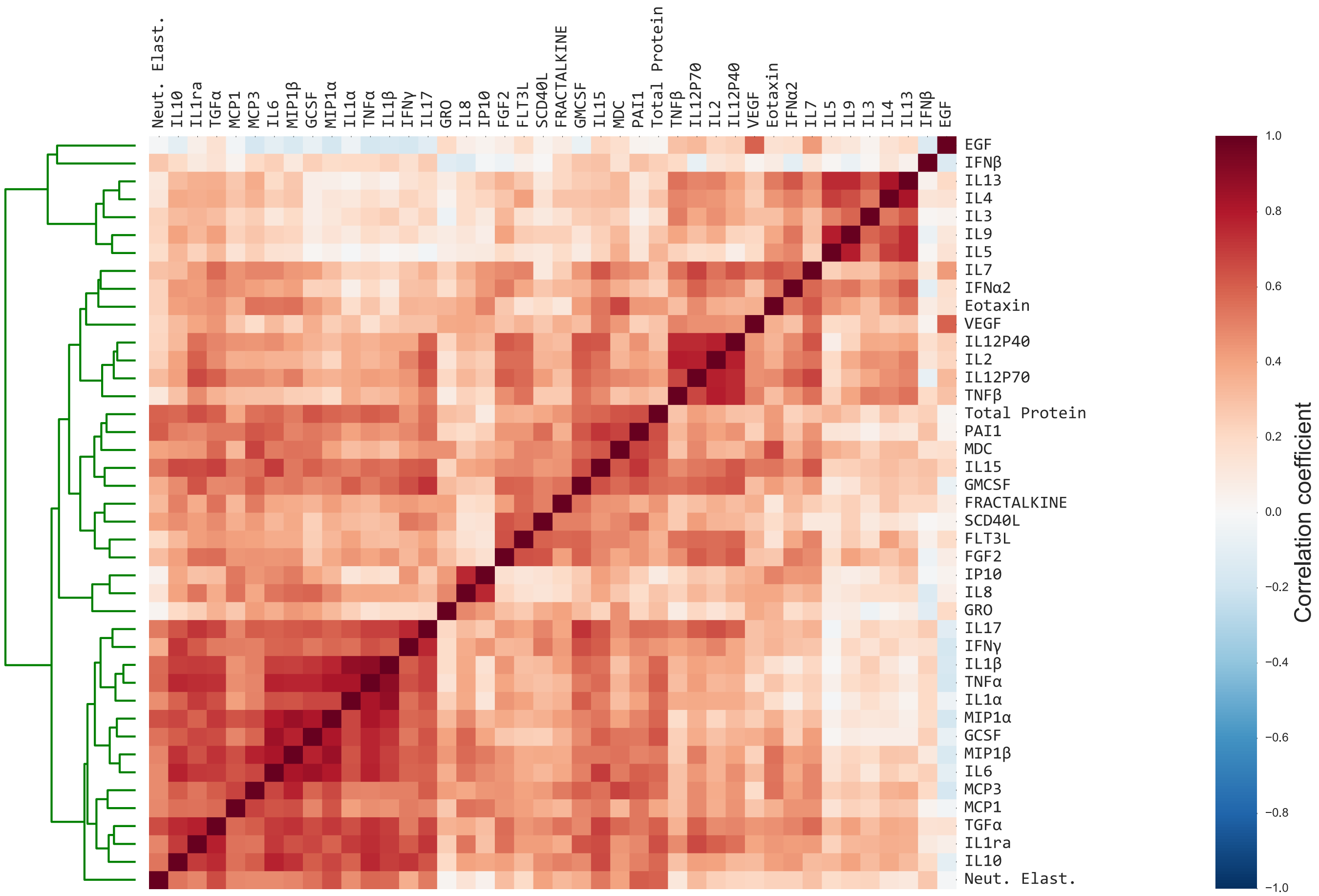

Figure S2B. ET cytokine correlation with mean

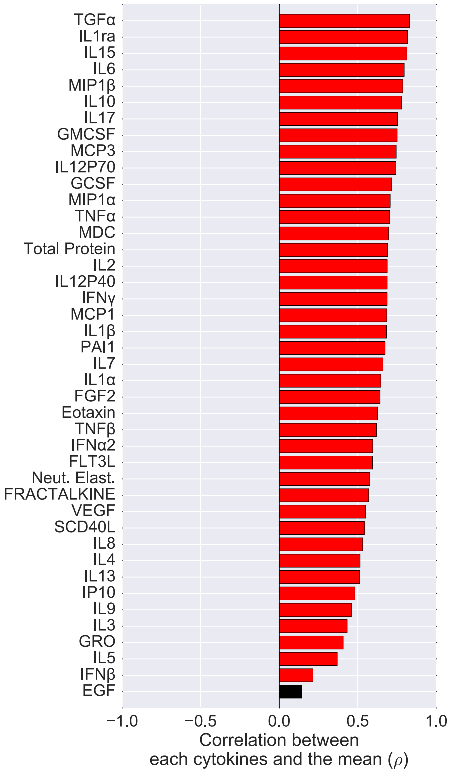

Figure S3. Cross-compartment cytokine correlation

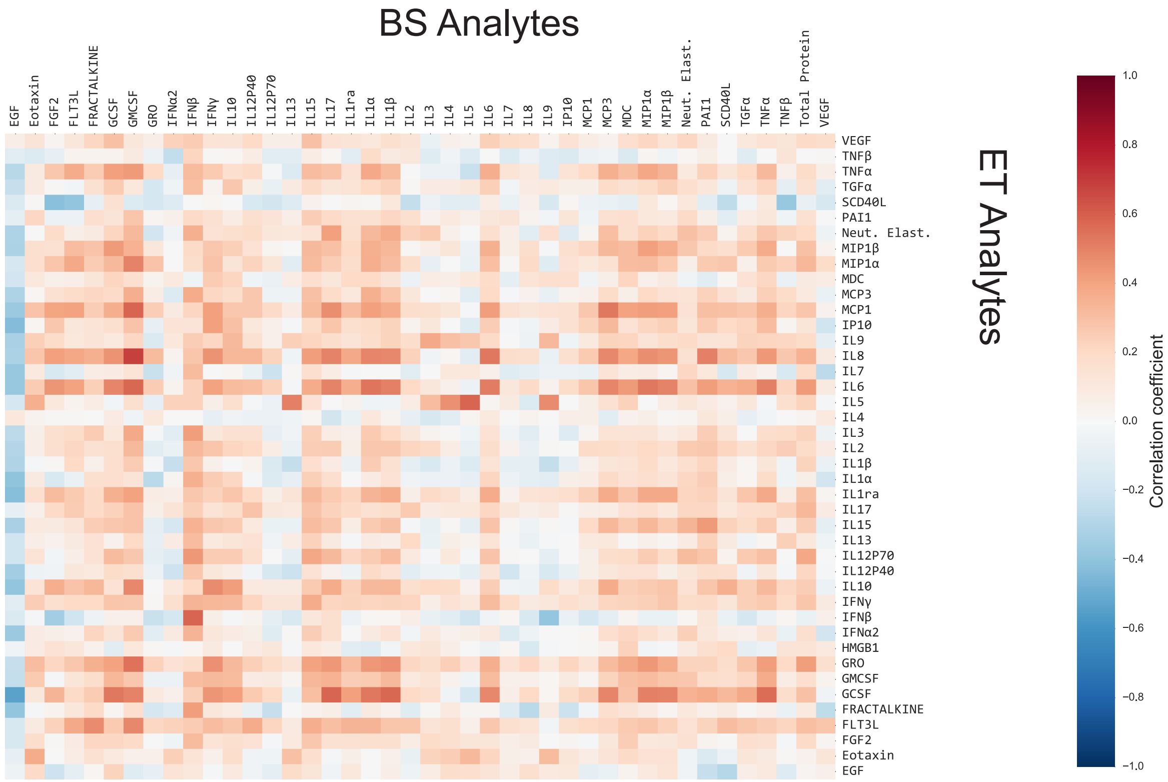

Figure S4. ET relative cytokine concentration correlations

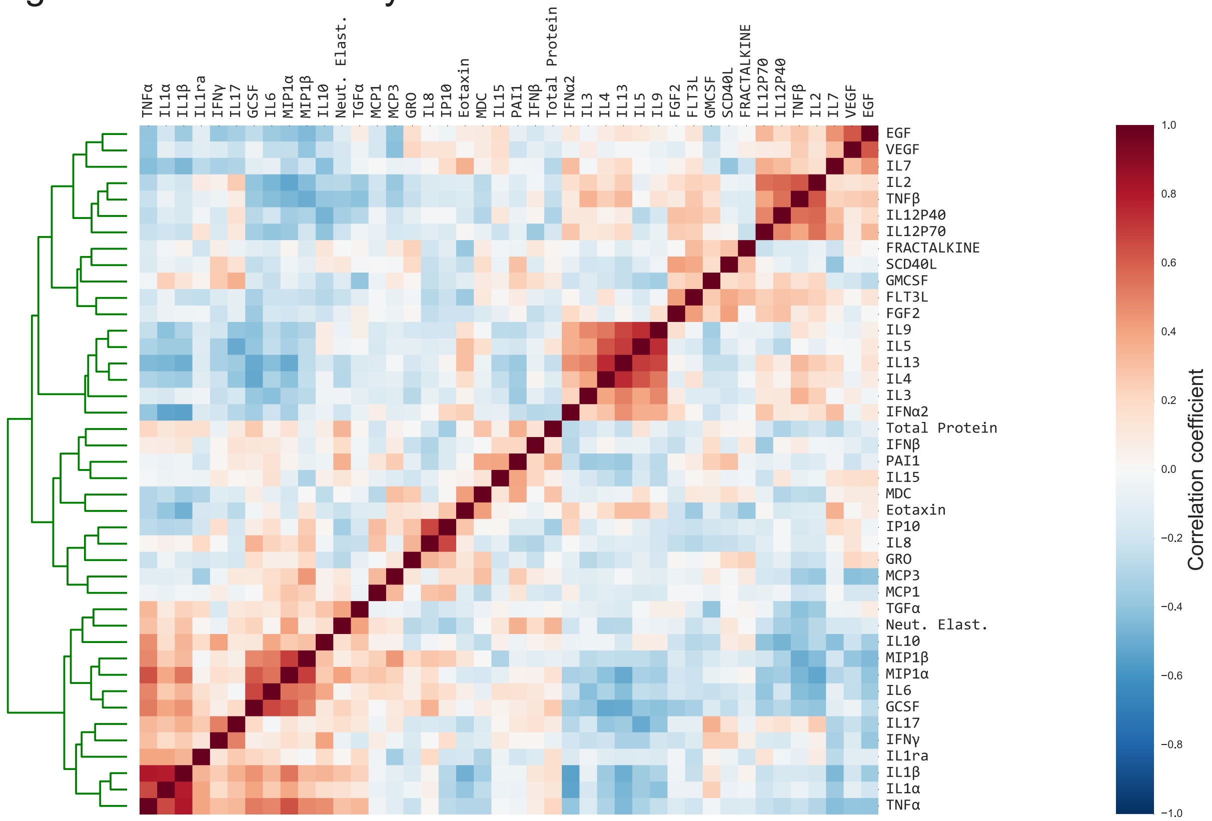

Figure S5. Modules of ET cytokines based on relative concentrations

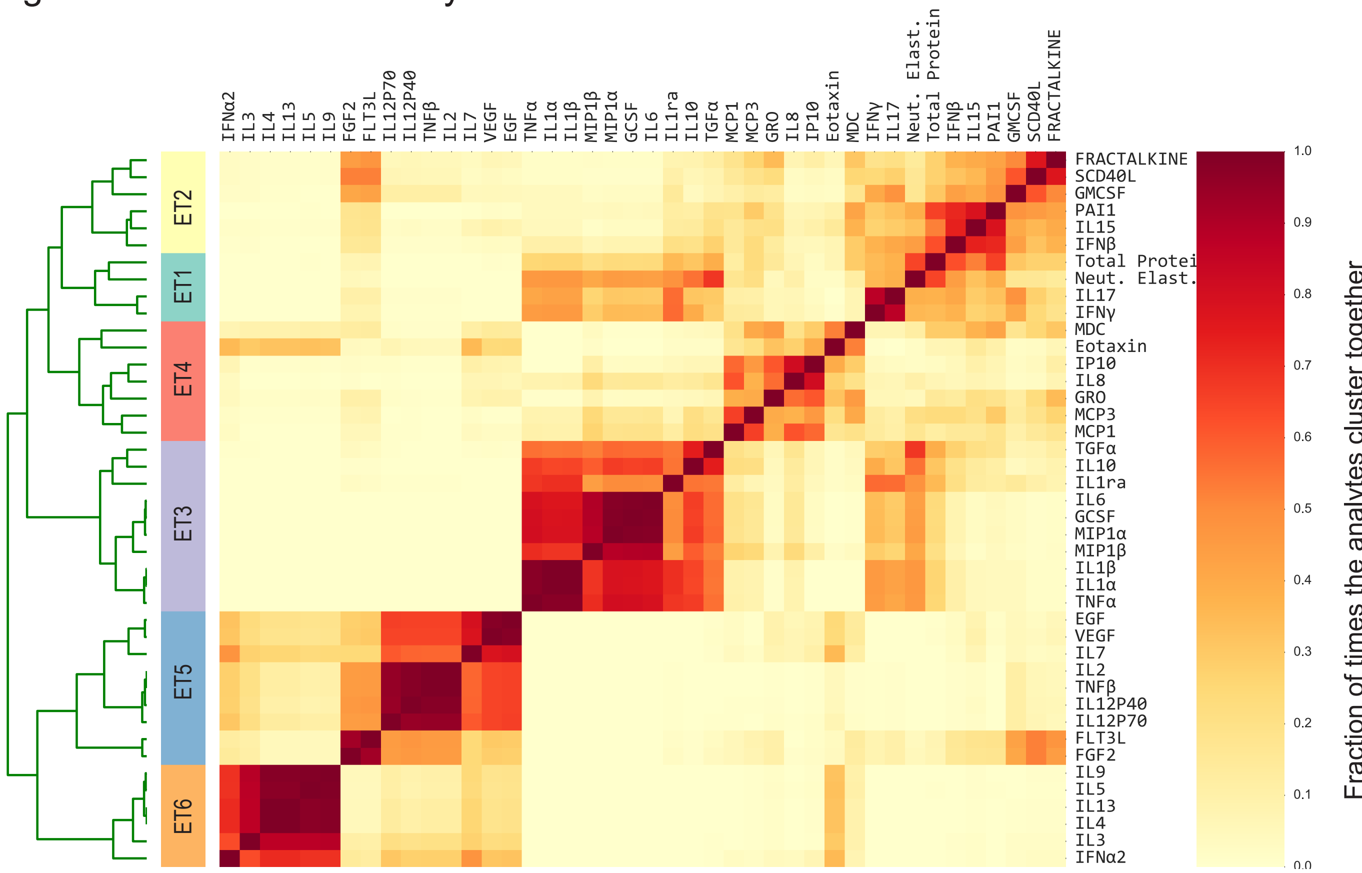

Figure S6. Associations with absolute cytokine concentrations

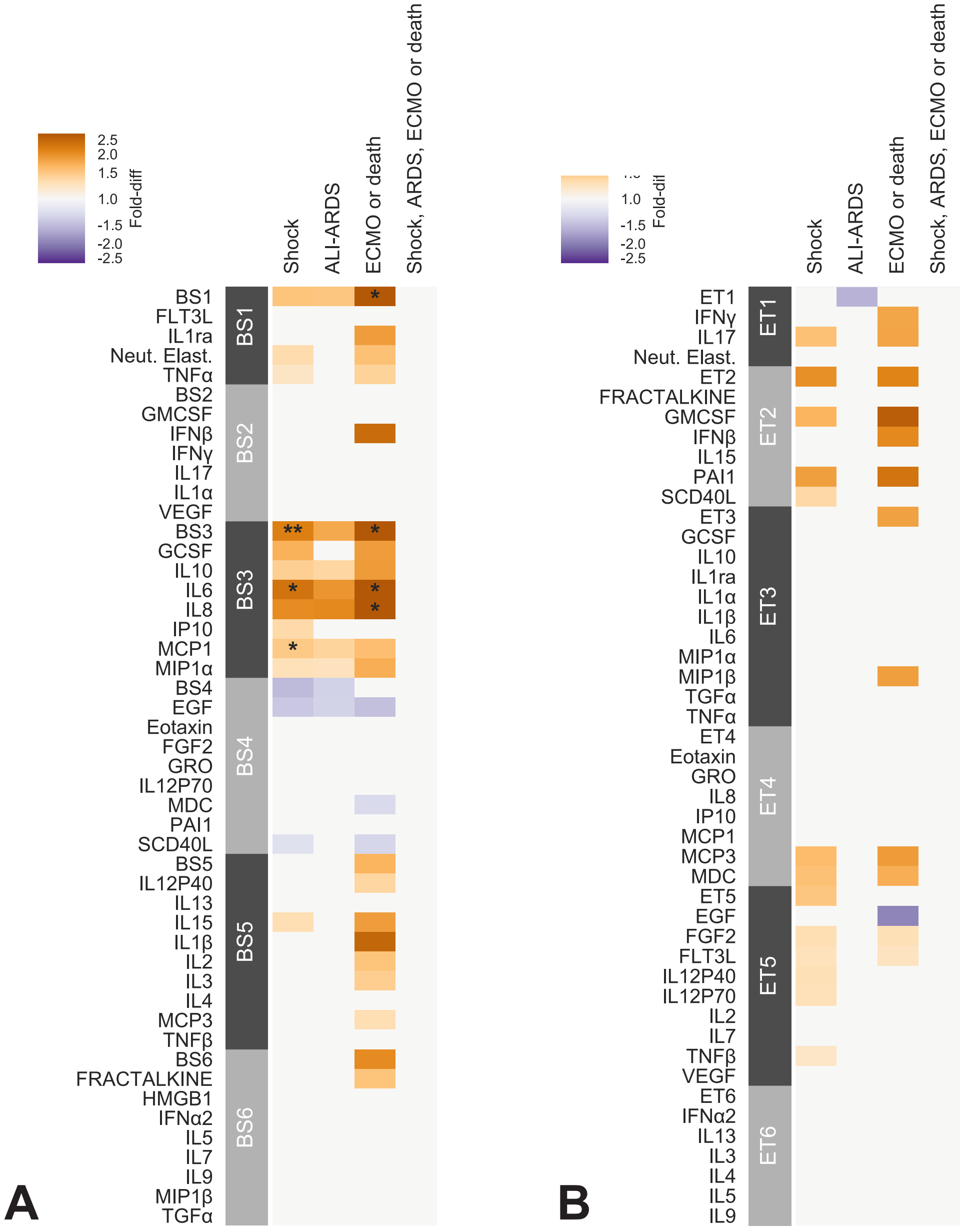

Figure S7. Immune profile of shock in the blood and lung

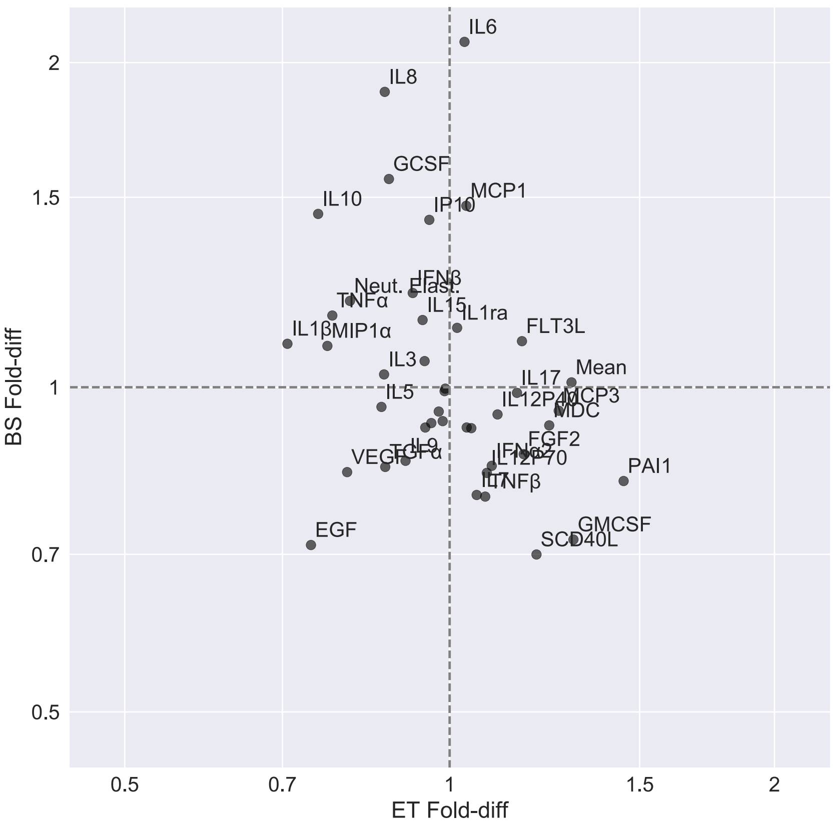

Table S1. Characteristics and clinical course of critically ill influenza positive patients (n=171)

	**Neither (n=70)**	**ALI/ARDS only (n=22)**	**Shock only (n=20)**	**Both (n=59)**	**P-Value**
**Male, N(%)**	41 (58.6)	12 (54.5)	13 (65)	36 (61)	0.910
**Hispanic, N(%)**	18 (25.7)	8 (36.4)	2 (10)	14 (23.7)	0.260
**Race, N(%)**					0.145
White	47 (67.1)	15 (68.2)	18 (90)	48 (81.4)	
Black	9 (12.9)	5 (22.7)	1 (5)	6 (10.2)	
Other	14 (20)	2 (9.1)	1 (5)	5 (8.5)	
**Age, years, median(IQR)**	5.6 (2.3, 8.9)	4.1 (1.8, 6.4)	7.3 (3.3, 9.5)	9.4 (5.7, 13.8)	*<0.001*
**Baseline Health Status***					
**Previously Healthy, N(%)**	42 (60)	15 (68.2)	12 (60)	36 (61)	0.920
Mild Chronic Respiratory	25 (35.7)	4 (18.2)	5 (25)	15 (25.4)	
Neurologic	3 (4.3)	3 (13.6)	5 (25)	9 (15.3)	
Other	3 (4.3)	0 (0)	1 (5)	12 (20.3)	
**Bacterial Pathogen Identified, N(%)****	23 (32.9)	10 (45.5)	4 (20)	28 (47.5)	0.098
**Support and Complications, N(%)**					
Mechanical Ventilation***					*<0.001*
None	14 (20)	0 (0)	1 (5)	0 (0)	
Non-Invasive Only	20 (28.6)	0(0)	2 (10)	0 (0)	
Any Invasive	36 (51.4)	22 (100)	17 (85)	59 (100)	
Myocarditis	0 (0)	0 (0)	0 (0)	3 (100)	*0.028*
Encephalitis	2 (2.9)	0 (0)	0 (0)	1 (1.7)	1.00
Extracorporeal Life Support	0 (0)	2 (9.1)	2 (10)	19 (32.2)	*<0.001*
**PRISM Score, median(IQR)**	3 (0, 6.8)	5.5 (3, 8)	8.5 (5, 14.8)	14 (9, 21)	*<0.001*
**VFDS, median(IQR)**	25.7 (23.4, 27.1)	20.5 (18.1, 22.9)	24.1 (21.5, 25.4)	16.1 (4.2, 19.5)	*<0.001*
**Duration of PICU stay, hours, median(IQR)**	99.5 (65, 139.8)	258 (185.2, 346)	163 (93.8, 229.2)	356 (231, 628.5)	*<0.001*
**Mortality, N(%)**	1 (1.4)	0 (0)	0 (0)	10 (16.9)	*<0.001*

***Some patients had more than one underlying medical condition.**

****Some patients had more than one bacterial pathogen identified.**

*****Some patients were on both non-invasive and invasive mechanical ventilation.**

Table S2. Identified bacterial and non-influenza viral pathogens in critically ill influenza positive patients (n=171)

	**Neither (n=70)**	**ALI/ARDS only (n=22)**	**Shock only (n=20)**	**Both (n=59)**	**P-Value**
**Bacterial Pathogen Identified, N(%)***	23 (32.9)	10 (45.5)	4 (20)	28 (47.5)	0.098
Staphylococcus aureus	10 (14.3)	4 (18.2)	1 (5)	22 (37.3)	*0.003*
Methicillin-sensitive *Staphylococcus aureus*	10 (14.3)	1 (4.5)	1 (5)	10 (16.9)	
Methicillin-resistant *Staphylococcus aureus*	0 (0)	3 (13.6)	0 (0)	12 (20.3)	
Streptococcus pneumoniae	4 (5.7)	1 (4.5)	0 (0)	4 (6.8)	
Streptococcus pyogenes	2 (2.9)	0 (0)	3 (15)	2 (3.4)	
Other	13 (18.6)	6 (27.3)	0 (0)	4 (6.8)	
**Non-Influenza Viral Pathogens Identified, N(%)***	7 (10)	3 (13.7)	4 (20)	5 (8.5)	0.820
Respiratory syncytial virus	3 (4.3)	1 (4.5)	1 (5)	0 (0)	
Adenovirus	2 (2.9)	1 (4.5)	0 (0)	1 (1.7)	
Rhinovirus	2 (2.9)	0 (0)	1 (5)	5 (8.5)	
Other	2 (2.9)	1 (4.5)	3 (15)	1 (1.7)	
**Influenza Type, N(%)****					*0.047*
Influenza A	61 (87.1)	20 (90.1)	18 (90)	41 (69.5)	
Influenza A H3	12 (17.1)	3 (13.6)	5 (25)	6 (10.2)	
Influenza A 2009 H1	36 (51.4)	12 (54.5)	7 (35)	29 (49.2)	
Influenza A Seasonal H1	3 (4.3)	4 (18.2)	1 (5)	1 (1.7)	
Influenza A – No Subtype	10 (14.3)	1 (4.5)	5 (25)	5 (8.5)	
Influenza B	10 (14.3)	2 (9.1)	2 (10)	18 (30.5)	

***Some patients had more than one bacterial and/or viral pathogen identified.**

****One patient was positive for influenza A 2009 H1 and influenza B.**

Table S3 Associations with modules of BS cytokines (relative concentrations)

Outcome	Module	OR	LL	UL	Fold-diff	pvalue	FWER	FDR
Shock	BS3	3.37	2.16	5.27	2.54	9.58e-08	1.74e-05	1.74e-05
Shock	BS4	0.426	0.29	0.628	0.484	1.56e-05	0.0028	0.000711
ALI-ARDS	BS3	2.15	1.49	3.1	1.96	4.81e-05	0.00846	0.00125
ECMO or death	BS4	0.429	0.272	0.676	0.407	0.000265	0.0458	0.00482
ECMO or death	BS3	2.33	1.45	3.75	2.32	0.000499	0.0853	0.00756
ALI-ARDS	BS4	0.57	0.404	0.805	0.595	0.00138	0.23	0.0148
Shock	BS1	1.69	1.2	2.39	1.63	0.00266	0.432	0.0217
Shock	BS6	0.604	0.433	0.842	0.621	0.00295	0.468	0.0223
ECMO or death	Mean	3.45	1.48	8	1.42	0.00398	0.629	0.029
ECMO or death	BS1	2.11	1.27	3.52	1.93	0.00416	0.653	0.0291
Ventilation	BS6	0.483	0.286	0.818	0.476	0.00679	1	0.0428
Ventilation	BS1	1.98	1.19	3.28	2.05	0.00823	1	0.0484
ECMO or death	BS2	0.592	0.385	0.909	0.576	0.0165	1	0.0784
ALI-ARDS	BS1	1.49	1.07	2.07	1.46	0.0172	1	0.0784
ALI-ARDS	BS6	0.676	0.49	0.933	0.686	0.0172	1	0.0784
Ventilation	BS3	1.74	0.978	3.08	1.65	0.0595	1	0.184

Table S4. Associations with modules of ET cytokines (relative concentrations)

Outcome	Module	OR	LL	UL	Fold-diff	pvalue	FWER	FDR
ECMO or death	ET2	3.59	1.83	7.03	3.38	0.000195	0.0339	0.00394
ECMO or death	ET6	0.373	0.163	0.85	0.515	0.019	1	0.0843
Shock	Mean	2.03	1.01	4.07	1.3	0.047	1	0.167
Shock	ET2	1.53	0.98	2.39	1.49	0.0616	1	0.187

Table S5. Associations with relative BS cytokine concentrations

Outcome	Analyte	OR	LL	UL	Fold-diff	pvalue	FWER	FDR
Shock	MCP1	11	4.18	29	1.41	1.19e-06	0.00142	0.00142
Shock	IL6	2.71	1.78	4.12	2.01	3.02e-06	0.00359	0.0018
ALI-ARDS	MCP1	6.37	2.64	15.4	1.33	3.77e-05	0.0446	0.00561
Shock	EGF	0.111	0.0387	0.32	0.699	4.56e-05	0.0538	0.00602
ALI-ARDS	IL8	2.51	1.59	3.97	1.87	7.53e-05	0.0888	0.00814
Shock	GCSF	2.61	1.61	4.22	1.58	9.92e-05	0.117	0.00889
ECMO or death	EGF	0.0376	0.00716	0.198	0.594	0.000107	0.126	0.00889
Shock	IL8	2.37	1.52	3.7	1.82	0.000138	0.163	0.0102
ALI-ARDS	IL6	2.06	1.41	3.03	1.72	0.000205	0.24	0.0123
ECMO or death	IL8	3.66	1.75	7.64	2.05	0.000571	0.665	0.0232
ECMO or death	VEGF	0.0928	0.024	0.359	0.706	0.000577	0.671	0.0232
ALI-ARDS	EGF	0.183	0.069	0.483	0.746	0.000613	0.712	0.0232
Shock	IP10	3.65	1.74	7.69	1.29	0.000645	0.746	0.0232
ECMO or death	IL6	2.74	1.52	4.96	2.04	0.000856	0.987	0.026
ECMO or death	IL4	0.00348	0.0001	0.121	0.826	0.00176	1	0.0367
ALI-ARDS	IFNα2	0.263	0.114	0.609	0.812	0.00184	1	0.037
ECMO or death	MDC	0.0964	0.022	0.423	0.726	0.00192	1	0.0376
Ventilation	IL8	2.14	1.32	3.47	2.22	0.00207	1	0.0385
Shock	IL12P70	0.347	0.176	0.683	0.779	0.0022	1	0.0396
ECMO or death	SCD40L	0.288	0.126	0.661	0.715	0.00328	1	0.0542
ECMO or death	IFNβ	1.94	1.25	3.03	1.95	0.00339	1	0.0553
Shock	TNFα	5.03	1.68	15	1.15	0.00384	1	0.061
ECMO or death	Mean	3.45	1.48	8	1.42	0.00398	1	0.0616
Ventilation	IL12P70	0.236	0.0866	0.642	0.674	0.00469	1	0.0662
Shock	FGF2	0.0883	0.0161	0.483	0.875	0.00514	1	0.0687
Shock	IL10	2.03	1.23	3.36	1.33	0.00586	1	0.0714
Shock	SCD40L	0.384	0.193	0.761	0.809	0.00608	1	0.0716
Shock	IFNα2	0.321	0.142	0.725	0.834	0.00628	1	0.0725
ALI-ARDS	VEGF	0.345	0.159	0.749	0.836	0.00715	1	0.0796
ECMO or death	MCP1	4.89	1.51	15.9	1.29	0.00813	1	0.086
ALI-ARDS	FGF2	0.113	0.0213	0.6	0.885	0.0105	1	0.102
Shock	IL15	3.08	1.29	7.37	1.25	0.0116	1	0.107
ECMO or death	IFNγ	0.463	0.253	0.848	0.641	0.0127	1	0.114
ECMO or death	GMCSF	0.516	0.303	0.877	0.617	0.0146	1	0.123
Shock	Neut. Elast.	1.9	1.13	3.18	1.28	0.0148	1	0.124
Ventilation	Neut. Elast.	2.62	1.19	5.74	1.51	0.0162	1	0.131
ALI-ARDS	IL10	1.82	1.11	2.98	1.28	0.0178	1	0.136
Shock	GRO	0.33	0.131	0.828	0.876	0.0182	1	0.136
Ventilation	MIP1β	0.219	0.0615	0.777	0.742	0.0187	1	0.136
ECMO or death	IL10	2.42	1.16	5.04	1.42	0.0188	1	0.136
Ventilation	IL6	2.02	1.1	3.71	1.73	0.0235	1	0.153
ALI-ARDS	MDC	0.313	0.114	0.863	0.876	0.0247	1	0.158
Shock	VEGF	0.42	0.196	0.899	0.862	0.0255	1	0.158
ECMO or death	IL1β	1.65	1.06	2.55	1.68	0.0256	1	0.158
Ventilation	IL15	7.61	1.27	45.6	1.44	0.0264	1	0.162
Shock	PAI1	0.344	0.132	0.898	0.891	0.0294	1	0.17
ECMO or death	IL15	3.88	1.14	13.1	1.32	0.0295	1	0.171
Shock	IL7	0.671	0.467	0.964	0.743	0.0307	1	0.174
ECMO or death	MIP1α	2.47	1.08	5.63	1.29	0.0313	1	0.176
ECMO or death	IL17	0.504	0.27	0.941	0.665	0.0314	1	0.176

Table S6. Associations with relative concentrations of ET cytokines

Outcome	Analyte	OR	LL	UL	Fold-diff	pvalue	FWER	FDR
ECMO or death	GMCSF	10.6	3.03	37.3	2	0.000222	0.26	0.0126
ECMO or death	EGF	0.27	0.12	0.607	0.489	0.00156	1	0.0361
ECMO or death	PAI1	4.84	1.62	14.5	1.74	0.00479	1	0.0662
Shock	FLT3L	19.4	2.35	161	1.17	0.00588	1	0.0714
Shock	PAI1	2.56	1.27	5.18	1.45	0.00867	1	0.0883
Shock	FGF2	8.73	1.58	48.3	1.17	0.013	1	0.114
ECMO or death	IFNβ	2	1.13	3.53	1.87	0.0172	1	0.135
ALI-ARDS	GMCSF	3.44	1.24	9.51	1.32	0.0173	1	0.135
ECMO or death	IL9	0.126	0.0226	0.696	0.669	0.0176	1	0.136
ALI-ARDS	VEGF	0.31	0.117	0.822	0.801	0.0186	1	0.136
ECMO or death	MCP3	4.57	1.29	16.2	1.49	0.0187	1	0.136
Shock	VEGF	0.316	0.119	0.839	0.804	0.0207	1	0.142
ECMO or death	IL13	0.176	0.04	0.772	0.699	0.0213	1	0.144
Shock	GMCSF	3.22	1.18	8.78	1.3	0.0224	1	0.148
ALI-ARDS	MCP3	2.88	1.12	7.43	1.3	0.0288	1	0.168
ALI-ARDS	TGFα	0.17	0.0345	0.843	0.857	0.03	1	0.172
Shock	SCD40L	3.77	1.12	12.7	1.2	0.0317	1	0.176

Table S7. Associations with modules of BS cytokines(absolute concentrations)

Outcome	Module	OR	LL	UL	Fold-diff	pvalue	FWER	FDR
Shock	BS3	2.52	1.68	3.79	2.14	8.98e-06	0.00162	0.000545
ECMO or death	BS3	2.6	1.66	4.07	2.88	2.89e-05	0.00515	0.00105
ECMO or death	BS1	2.98	1.75	5.07	2.73	5.55e-05	0.00971	0.00126
ALI-ARDS	BS3	1.86	1.3	2.65	1.76	0.000612	0.104	0.00857
ECMO or death	BS6	2.04	1.28	3.24	2.03	0.00252	0.411	0.0217
Shock	BS1	1.57	1.12	2.19	1.53	0.00846	1	0.0484
Shock	BS4	0.644	0.464	0.894	0.657	0.00852	1	0.0484
ALI-ARDS	BS1	1.55	1.11	2.16	1.51	0.0102	1	0.0562
Ventilation	BS1	1.89	1.09	3.28	1.84	0.0224	1	0.097
ECMO or death	BS5	1.6	1.06	2.42	1.67	0.0266	1	0.109
Ventilation	BS3	1.93	0.998	3.74	1.68	0.0506	1	0.167
ALI-ARDS	BS4	0.743	0.542	1.02	0.748	0.0657	1	0.193

Table S8. Associations with modules of ET cytokines (absolute concentrations)

Outcome	Module	OR	LL	UL	Fold-diff	pvalue	FWER	FDR
Shock	ET2	2.15	1.32	3.5	1.98	0.00205	0.338	0.0207
ECMO or death	ET2	2.35	1.21	4.55	2.1	0.0114	1	0.0611
ALI-ARDS	ET1	0.618	0.398	0.961	0.634	0.0327	1	0.128
ECMO or death	ET3	1.95	1.04	3.65	1.83	0.0377	1	0.143
Shock	ET5	1.53	0.996	2.34	1.51	0.0525	1	0.17

Table S9. Associations with absolute concentrations of BS cytokines

Outcome	Analyte	OR	LL	UL	Fold-diff	pvalue	FWER	FDR
ECMO or death	SCD40L	0.382	0.17	0.858	0.766	0.0198	1	0.14
ECMO or death	IL12P40	2.37	1.12	5.01	1.36	0.0237	1	0.153
Shock	MIP1α	1.73	1.07	2.79	1.27	0.0249	1	0.158
ECMO or death	MCP3	4.63	1.21	17.6	1.29	0.025	1	0.158
ALI-ARDS	MIP1α	1.67	1.04	2.69	1.25	0.0349	1	0.188
Shock	IL15	1.89	1.04	3.44	1.29	0.0367	1	0.194

Table S10. Associations with absolute concentrations of ET cytokines

Outcome	Analyte	OR	LL	UL	Fold-diff	pvalue	FWER	FDR
ECMO or death	GMCSF	4.34	1.84	10.3	2.61	0.000822	0.949	0.026
Shock	FLT3L	17.1	3.06	95.8	1.25	0.00123	1	0.0305
ECMO or death	PAI1	4.29	1.73	10.6	2.27	0.00167	1	0.0362
Shock	PAI1	2.47	1.4	4.35	1.85	0.00175	1	0.0367
Shock	FGF2	8.56	2.08	35.2	1.29	0.0029	1	0.0501
ECMO or death	EGF	0.306	0.14	0.67	0.513	0.00307	1	0.0514
Shock	GMCSF	3.11	1.41	6.82	1.67	0.00475	1	0.0662
Shock	SCD40L	4.64	1.58	13.6	1.34	0.00513	1	0.0687
ECMO or death	IFNβ	2.1	1.21	3.65	2.04	0.00868	1	0.0883
Shock	MDC	2.14	1.21	3.78	1.56	0.00901	1	0.0902
Shock	MCP3	2.31	1.23	4.33	1.6	0.0091	1	0.0903
ECMO or death	MCP3	3.09	1.26	7.63	1.88	0.0141	1	0.121
ECMO or death	MDC	2.73	1.19	6.27	1.73	0.0179	1	0.136
ECMO or death	IFNγ	2.18	1.13	4.22	1.79	0.0204	1	0.141
Shock	IL12P40	2.74	1.16	6.46	1.28	0.0211	1	0.143
Shock	IL17	1.69	1.06	2.68	1.56	0.0271	1	0.164
Shock	IL12P70	2.55	1.11	5.85	1.27	0.0276	1	0.166
ECMO or death	FLT3L	15.7	1.34	184	1.24	0.0282	1	0.166

Table S11. Validation of BS modules

Outcome	Module	N	OR	LL	UL	Fold-diff	pvalue
ECMO or death	BS4	73	0.146	0.0516	0.411	0.229	0.00027
Shock	BS3	73	3.11	1.65	5.84	2.49	0.000425
ALI-ARDS	BS3	73	2.82	1.53	5.19	2.36	0.000895
ECMO or death	BS3	73	4.88	1.87	12.7	3.26	0.00122
ALI-ARDS	BS4	73	0.532	0.319	0.885	0.549	0.0152
Shock	BS4	73	0.598	0.364	0.982	0.614	0.0423

Table S12. Biomarker development with absolute analyte concentrations

	Development			Validation		
	**AUC**	**Sensitivity**	**Specificity**	**AUC**	**Sensitivity**	**Specificity**
**LASSO**	0.75	0.53	0.82	0.67	0.42	0.70
**SVC**	0.76	0.51	0.86	0.69	0.46	0.77
**GBMC**	0.73	0.40	0.86	0.75	0.42	0.81
**PRISM**	0.84	0.61	0.87	0.70	0.46	0.75

Table S13. Distribution of enrollment by study site

	Number of PICUs with *X* patients enrolled, by cohort		
**Number of patients (*X*)**	**Development cohort**	**Validation cohort**	**Both cohorts**
**1**	5	11	8
**2**	4	2	2
**3**	6	4	5
**4**	2	4	4
**5**	2	2	0
**6**	2	0	1
**7**	4	2	3
**8**	1	0	0
**9**	1	1	1
**10**	0	0	4
**11**	1	0	2
**13**	0	0	1
**16**	1	0	1
**18**	1	0	0
**20**	1	0	0
**23**	0	0	1
**25**	0	0	1
**29**	0	0	1
